# UV-Induced Colour Changes in Bio-Based Coating Formulations Containing Turmeric and Madder Studied by EPR and FTIR

**DOI:** 10.3390/molecules31213930

**Published:** 2026-11-01

**Authors:** Wojciech Żyłka, Barbara Pilch-Pitera, Ireneusz Stefaniuk

**Affiliations:** 1 Faculty of Exact and Technical Sciences, Institute of Materials Engineering, University of Rzeszow, Pigonia 1, 35-310 Rzeszow, Poland; istefaniuk@ur.edu.pl; 2 Department of Polymers and Biopolymers, Faculty of Chemistry, Rzeszów University of Technology, ul. Powstańców Warszawy 6, 35-959 Rzeszów, Poland

**Keywords:** natural colorants, bio-based coatings, UV-responsive coatings, electron paramagnetic resonance, photopolymerization, madder, turmeric

## Abstract

The study investigated the mechanism of colour change in coating formulations containing natural colorants—madder (Rubia tinctorum) and turmeric (Curcuma longa)—under UV irradiation using electron paramagnetic resonance (EPR) and Fourier-transform infrared spectroscopy (FTIR). Samples based on Epidian^®^ 5 epoxy resin and Polimal^®^ 109 unsaturated polyester resin containing radical and cationic photoinitiators were examined. EPR analysis revealed the involvement of transition metal ions (Fe^3+^, Cu^2+^) and organic radicals in the photo-oxidation processes. Characteristic signals at g ≈ 4.3, g = 2.2–2.3 and g = 2.001 were attributed to Fe^3+^, Cu^2+^ and aromatic radicals, respectively. UV irradiation caused the formation of oxygen- and phenoxyl-type radicals in turmeric-containing epoxy coatings and phenoxyl and semiquinone radicals in madder-containing coatings. The colour changes correlated with structural transformations identified by FTIR spectroscopy, while polyester coatings showed slower colour-change dynamics. The results confirm the usefulness of EPR and FTIR spectroscopy for analyzing UV-induced processes in polymer coating formulations containing plant-derived colorants and provide insight into the photochemical stability of bio-based components considered for more environmentally friendly coating materials.

## 1. Introduction

Industrially cultivated plants have considerable potential for the development of sustainable materials, including plastics, composites, and protective or decorative coatings. In this context, turmeric and madder are promising plant-derived sources of natural colourants that may be used as functional additives in paint coatings. Their chromophoric compounds can undergo UV-induced transformations, leading to visible colour changes that depend on the wavelength and duration of irradiation. Such behaviour creates the possibility of developing bio-based UV-responsive coating systems as an alternative to coatings modified with conventional synthetic colourants. The increasing interest in bio-based and biodegradable polymeric materials has also stimulated research on plant-derived functional additives capable of partially replacing conventional synthetic components. In this context, understanding the stability and photochemical behaviour of natural additives in polymer matrices is relevant to the development of more sustainable polymer-based materials and coatings. The mechanisms responsible for UV-induced colour changes are often associated with photochemical reactions, including the formation of radical species and paramagnetic centres. These processes can be effectively investigated using Electron Paramagnetic Resonance (EPR) spectroscopy. EPR is a highly sensitive spectroscopic technique that enables the detection and analysis of paramagnetic centres, such as free radicals and transition-metal ions [1].

The presence of an unpaired electron makes radicals key species in photochemical processes [2], oxidation, and the degradation of organic dyes. The literature emphasizes that the g-factor, determined from the position of the resonance line, provides information about the chemical nature of the active centre, whereas the intensity and shape of the signal reflect its concentration and interactions with the surrounding environment [3,4,5]. Natural plant-derived colorants, such as madder (Rubia tinctorum) and turmeric (Curcuma longa), have been used for centuries in the dyeing of textiles and wood, as well as in art conservation [6,7]. The main chromophores in madder are anthraquinone derivatives, including alizarin, purpurin, and ruberythric acid, which are responsible for its intense red colour but also exhibit susceptibility to UV radiation [8]. Under UV irradiation, these compounds may undergo photochemical transformations involving radical intermediates. EPR spectroscopy is widely used to investigate organic radical species, including phenoxyl- and semiquinone-type radicals [9,10,11,12,13].

As a consequence, gradual darkening of the colour occurs [14]. Turmeric, in turn, contains curcumin, a polyphenolic pigment with an intense yellow colour and antioxidant properties [15,16]. Although curcumin is valued for its biological activity, it readily undergoes photochemical reactions under UV irradiation. This process leads to the formation of oxygen- and phenoxyl-type free radicals, which can be characterized by EPR spectroscopy. In studies on the application of natural dyes and pigments in paints, photoinitiators also play an important role, as they enable controlled polymerization of the polymer matrix under UV exposure. Both radical photoinitiators (e.g., Irgacure 651) and ionic photoinitiators (e.g., triarylsulfonium, phosphonium, pyridinium, or iodonium salts of strong acids such as HSbF6, HAsF6, HPF6, and HBF4) are of key importance, since upon absorption of UV light they generate reactive radicals or Brønsted acids that initiate polymer crosslinking processes [17,18,19]. The use of such systems makes it possible to obtain photocurable paints in which changes in pigment colour are coupled with chemical processes occurring in the polymer matrix [20,21,22]. Spectroscopic EPR studies of paints containing madder and turmeric therefore make it possible to simultaneously monitor the dynamics of the formation and decay of organic radicals in the colorants, the activity of photoinitiators, and the crosslinking process of the epoxy or polyester resin. Analysis of these processes provides not only information on the mechanism of photodegradation of natural colorants, but also allows evaluation of their suitability for photocurable paint formulations, which constitutes an important step toward the development of more sustainable, bio-based protective and decorative polymer materials [14,22]. Colour changes in natural colorants during UV exposure are usually associated with radical reactions that result in darkening or fading of the sample. A key stage of these transformations is the formation of stable aromatic free radicals [23].

High-spin Fe^3+^ ions in rhombic environments exhibit characteristic EPR signals around g ≈ 4.3 [24]. Phenoxyl radicals exhibit EPR signals near g ≈ 2.004 [9,25]. Studies of radical–copper systems have demonstrated that organic radical and metal-associated paramagnetic contributions may coexist [26], whereas semiquinone radicals exhibit resonance signals close to the free-electron g value [11,27,28]. Therefore, broad signals in the g ≈ 2.2–2.3 region cannot be unambiguously assigned to Cu^2+^ ions based solely on their resonance positions.

Curcumin and its derivatives are capable of forming oxygen- and phenoxyl-type radicals already in the early stages of irradiation, which are observed as EPR signals in the region of g ≈ 2.005 [29]. In madder, by contrast, photo-oxidation processes occur gradually, leading to the formation of phenoxyl and semiquinone radicals associated with anthraquinone chromophores [8,30,31,32,33]. Despite these reports, comprehensive and comparative EPR studies of paints containing natural colorants in polymer matrices subjected to UV irradiation remain scarce. Therefore, the aim of the present study was to investigate the mechanism of colour changes in paints containing madder and turmeric under UV irradiation using EPR spectroscopy as a direct tool for the detection of radicals and metallic centres. These investigations were complemented by FT-IR spectroscopy and colour measurements in order to explain in detail the processes occurring within the chemical structure of the coating. Examination of these processes makes it possible to assess the behaviour of colorants and coating formulations differing in chemical structure and curing mechanism under UV irradiation. Understanding this mechanism is important because it may help control colour changes by selecting the appropriate UV irradiation wavelength. The obtained results may also support the rational selection of plant-derived functional additives for emerging sustainable and partially bio-based polymer systems.

## 2. Results and Discussion

The aim of the present study was to investigate the mechanisms of chemical transformations responsible for colour changes in coating formulations containing either madder extract (*Rubia tinctorum*) or turmeric upon UV irradiation.

To achieve this objective, the following coating formulations were prepared:

➢ Formulation 1 contained madder extract as the colorant (madder red dye extract), the ionic photoinitiator triarylsulfonium hexafluoroantimonate, and Epidian 5 epoxy resin.
➢ Formulation 2 contained powdered turmeric as the colorant, the ionic photoinitiator triarylsulfonium hexafluoroantimonate, and Epidian 5 epoxy resin.
➢ Formulation 3 contained madder extract as the colorant, a radical photoinitiator, and Polimal 109 unsaturated polyester resin.

Madder extract is a traditional plant-derived colorant (madder red dye extract) containing mainly anthraquinone chromophores, including alizarin, which may undergo transformations under UV irradiation. Turmeric is likewise used as a yellow–orange polyphenolic plant-derived colorant containing curcumin. As the polymer matrix, a low-molecular-weight, low-viscosity Epidian^®^ 5 epoxy resin based on bisphenol A diglycidyl ether (DGEBA) and epichlorohydrin was used. Epoxy coatings are characterized by very good adhesion to substrates, as well as high chemical resistance and good dielectric properties. For its crosslinking, a cationic photoinitiator, triarylsulfonium hexafluoroantimonate, was employed. The cationic mechanism is less susceptible to oxygen inhibition and enables deeper curing of the coating, including so-called dark curing, which makes this system particularly useful for analysing the stability and colour changes of colorants in epoxy resins [18,34,35]. The photoinitiator used to initiate the cationic polymerization reaction, triarylsulfonium hexafluoroantimonate, belongs to the group of onium salts. Upon excitation by UV light, this compound undergoes photoionization, leading to the formation of strong Brønsted acids, which initiate epoxy ring opening and further cationic polymerization. Onium salts are widely used in the crosslinking of epoxy resins [18,34,36]. The second type of resin was Polimal^®^ 109, a conventional unsaturated polyester resin dissolved in styrene and containing C=C double bonds, which readily undergo copolymerization with styrene via a radical mechanism. Owing to this, the resin is widely used in radical-initiated photocurable systems, including laminates and coatings. To initiate the polymerization of Polimal^®^ 109 resin, the radical photoinitiator Irgacure 651 was used, enabling both the polymerization process and the monitoring of the degradation of natural colorants under UV irradiation [17,37,38]. Irgacure 651 (2,2-dimethoxy-2-phenylacetophenone) is a classical type I radical photoinitiator which, upon absorption of UV radiation, undergoes homolytic cleavage of a C–C bond, leading to the formation of benzyl and acyl radicals. The resulting radicals effectively initiate the free-radical polymerization of unsaturated bonds [5,37,38]. The combination of these two types of matrices, polyester and epoxy, made it possible to compare the behaviour of natural colorants during radical and cationic photopolymerization, thereby reflecting the diverse conditions encountered in industrial practice. The use of initiators operating through different polymerization mechanisms enabled comparison of the behaviour of the colorants in both radical and cationic polymerization environments. These differences are significant because the initiation mechanism governs the crosslinking kinetics, radical stability, and the course of colour changes under UV irradiation.

**Figure 1:**
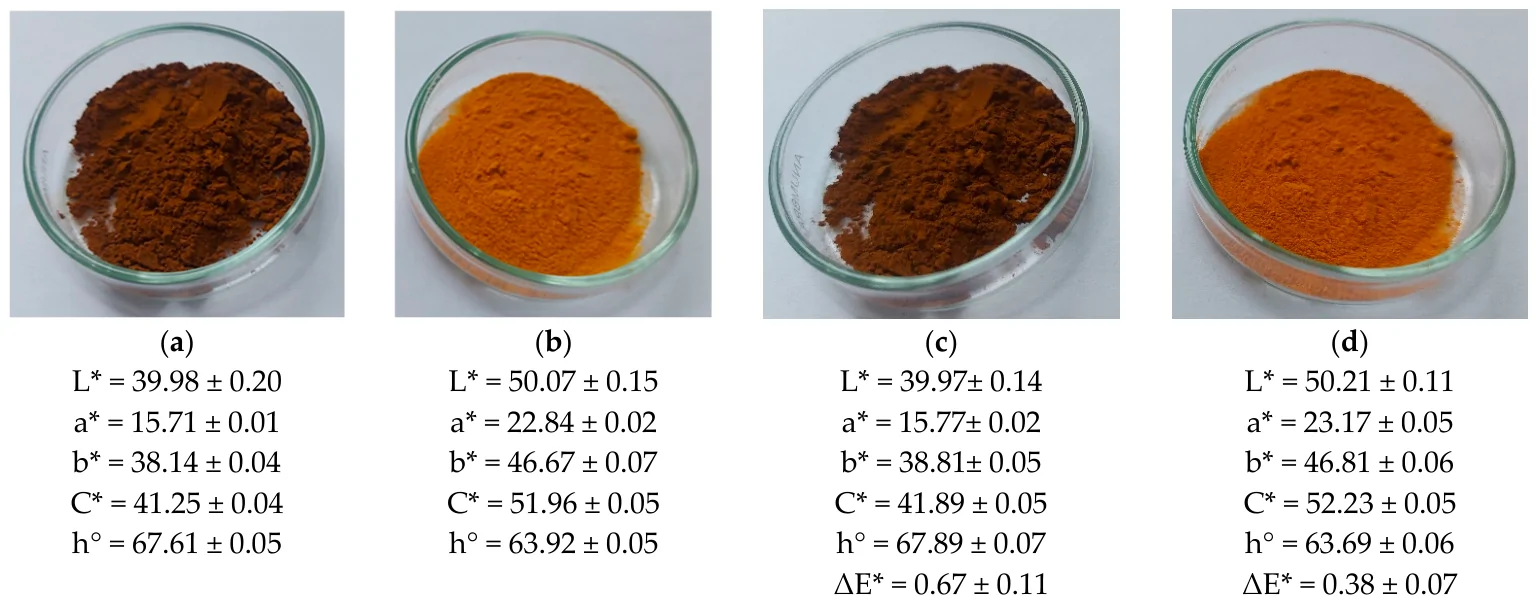
Madder and turmeric before and after heating in an oven at 100 °C for 1 h: (**a**) madder before heating, (**b**) turmeric before heating, (**c**) madder after heating, and (**d**) turmeric after heating.

To evaluate the effect of temperature on the colour change of the mixtures during curing, the samples were placed in an oven at 100 °C (Figure 1).

After 60 min, no colour change was observed in any of the samples. The samples were subsequently exposed to UV radiation, which enabled observation of the colour changes and analysis of the photochemical processes occurring in the formulations. This observation was verified by additional spectrophotometric colour measurements performed before and after heating at 100 °C for 60 min. The calculated colour differences were ΔE* = 0.67 ± 0.11 for madder and ΔE* = 0.38 ± 0.07 for turmeric, indicating no significant colour changes after heating.

### 2.1. EPR Spectroscopy

To investigate the behaviour of the colorants themselves in the absence of resins, the study began with the recording of EPR spectra of turmeric and madder samples, before and after UV irradiation, in mixtures containing the colorants and photoinitiators. Prior to UV irradiation, EPR measurements of the neat Epidian^®^ 5 and Polimal^®^ 109 resins were performed. No detectable EPR signals were observed under the applied measurement conditions.

#### 2.1.1. EPR Study of Madder

**Figure 2:**
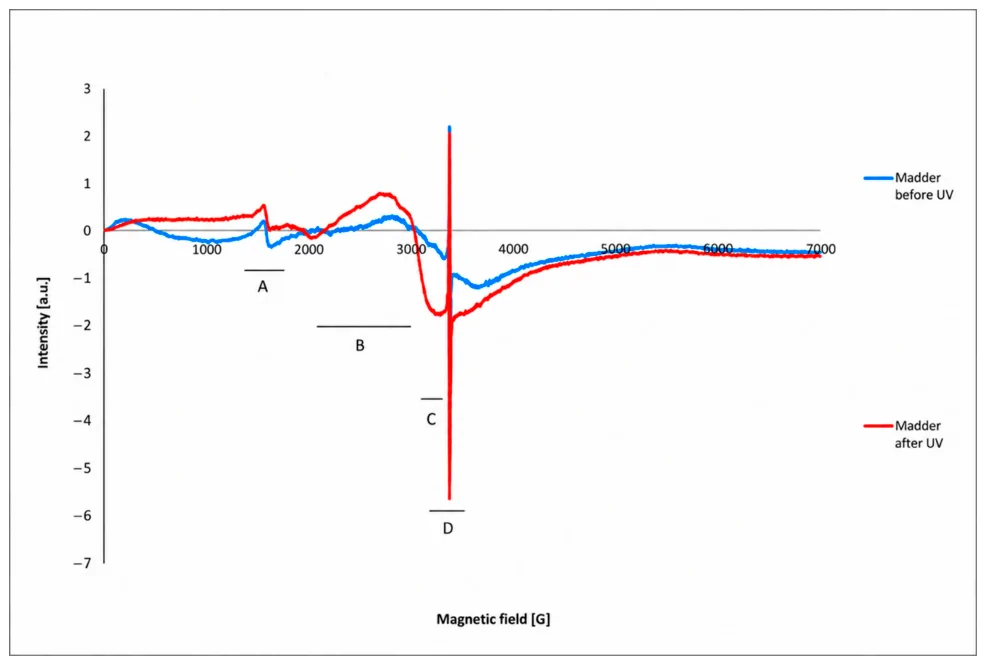
EPR spectra of the madder–photoinitiator mixture before UV irradiation (Capillary 7, blue line) and after UV irradiation (red line).

Figure 2 shows the EPR spectra of madder extract before and after UV irradiation. Four characteristic regions, denoted as lines A–D, were distinguished in the spectra, and their parameters are summarized in Table 1.

**Line A** (*B*_0_ ≈ 1585 G, *g* = 4.25)—in this region, a signal characteristic of high-spin Fe^3+^ ions in rhombic symmetry is observed [24]. After UV irradiation, the *g* value remains unchanged (4.25), indicating that the nature of the paramagnetic centre does not change. Slight differences in signal amplitude (intensity: 0.3048 vs. 0.3963 a.u.) and linewidth (Bpp ≈ 66 G) may indicate migration of a small fraction of metal ions or changes in their local environment [1,24,39,40].

**Line B** (*B*_0_ ≈ 3072 G, *g* = 2.19)—a broad signal appears in the spectral region around *g* ≈ 2.19 only after UV irradiation, indicating the presence of transition-metal centres, including low-spin Fe^3+^, which are typical of natural plant-derived colorants. The width and asymmetry of line B suggest overlapping signals from several metallic centres, which is consistent with the fact that natural plant colorants may contain trace amounts of various metal ions, including Fe, Mn, and Cu [41,42]. Numerous studies have shown that compounds with anthraquinone structures (e.g., alizarin), lignins, and other plant pigments may form complexes with transition-metal ions and consequently generate EPR signals, even when these metals are present only in trace amounts [41,42]. Therefore, this signal should be interpreted as the combined contribution of several paramagnetic centres, primarily low-spin Fe^3+^ and possibly also Cu^2+^. Its assignment exclusively to Cu^2+^ ions is not justified, because the line is too broad and does not exhibit the typical asymmetry or structure characteristic of Cu^2+^ complexes. The absence of hyperfine features does not allow the contribution of Co^2+^ to be excluded, since at room temperature the Co^2+^ signal may be poorly resolved or even undetectable due to its large linewidth and low intensity. The appearance of this line only after UV irradiation indicates photochemical activation of paramagnetic centres and their possible involvement in redox reactions [43].

**Line C** (region between lines B and D)—this region is difficult to resolve unambiguously because the tail of signal B overlaps with the onset of signal D. Its width and shape rather indicate overlapping signals from transition metals, including Fe^3+^ in different coordination states. It should therefore be regarded as a composite region, which does not exhibit a single maximum and does not allow unambiguous determination of spectroscopic parameters.

**Line D** (B_0_ ≈ 3370–3372 G, g = 2.001)—the most stable signal, observed both before and after UV irradiation, was assigned to organic radicals with semiquinone or phenoxyl structures [41,42,44,45]. The narrow line at g ≈ 2.001 (B_0_ ≈ 3370 G) is characteristic of semiquinone- or phenoxyl-type organic radicals formed as a result of UV irradiation of the anthraquinone colorants present in madder [25,26]. The value g = 2.001 represents the measured response of overlapping radical contributions. Considering the estimated error of ±0.003, the resulting g-factor interval partially overlaps with the literature range of 2.003–2.006. After UV irradiation, the signal becomes clearly more intense (intensity: 5.9171 vs. 4.6483 a.u.) and slightly broader (Bpp ≈ 6.8 vs. 6.1 G), which indicates an increase in the number of stable free radicals. This is the main and most characteristic EPR signal of madder extract. The increased intensity of this signal after UV irradiation confirms that free-radical generation is the principal mechanism affecting the colour changes in the sample.

#### 2.1.2. EPR Study of Turmeric

**Figure 3:**
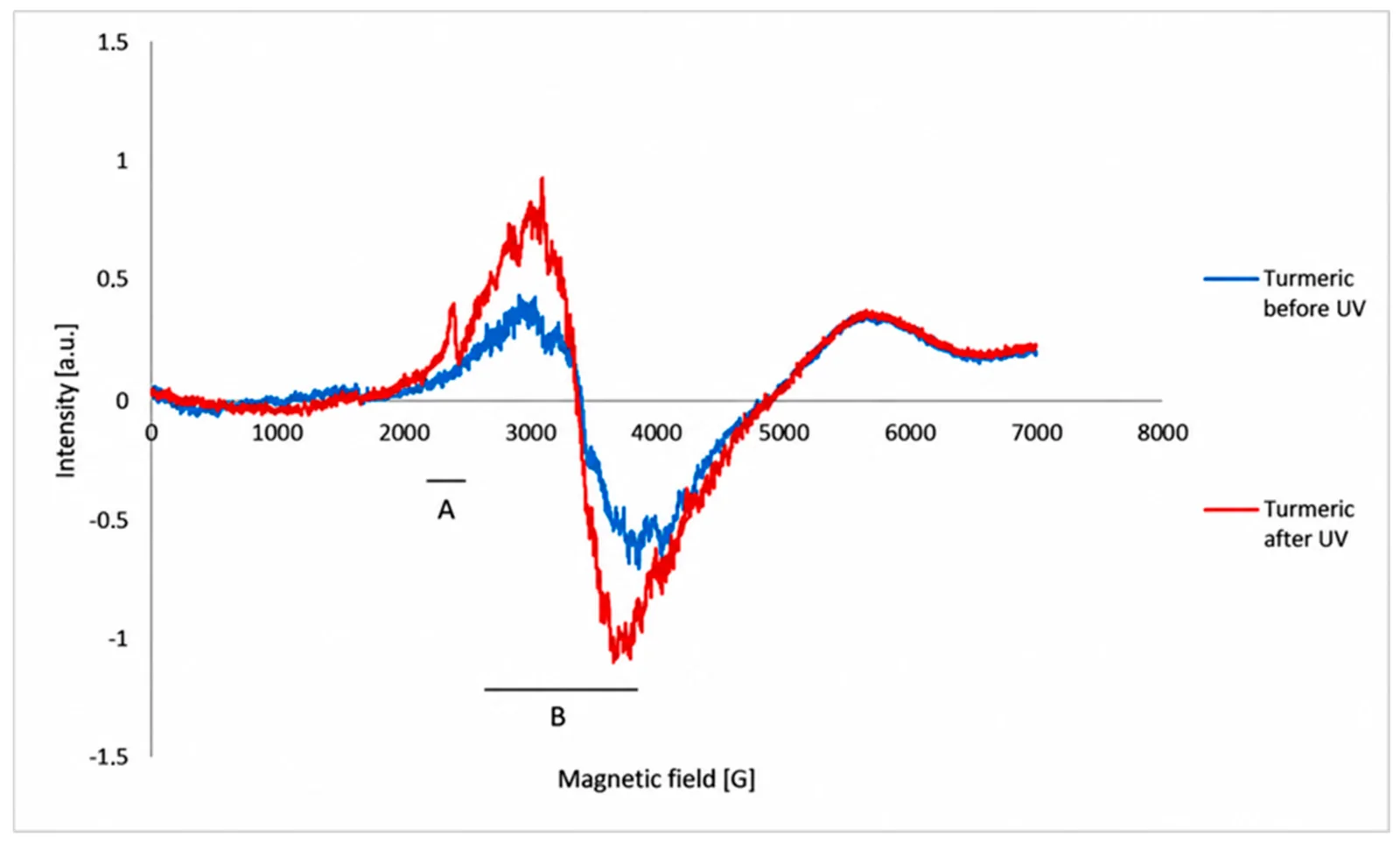
EPR spectra of the turmeric–photoinitiator mixture before UV irradiation (blue line) and after UV irradiation (red line).

The EPR spectra of the turmeric mixture with the ionic photoinitiator triarylsulfonium hexafluoroantimonate show the presence of two characteristic signals (Figure 3, Table 2).

**Line A**—After UV irradiation, a signal appeared at B_0_ ≈ 2409 G, g ≈ 2.92, and Bpp ≈ 38 G. This signal may be attributed to low-spin Fe^3+^ (S = 1/2) centres in a distorted (rhombic) coordination environment and may indicate their photochemical activation or changes in their local environment [24,46]. However, the involvement of redox processes cannot be unequivocally confirmed by EPR alone. In low-spin Fe^3+^ complexes with rhombic symmetry, a resonance in this spectral region may correspond to the g_z component of the anisotropic g tensor.

**Line B** (B_0_ ≈ 3404–3444 G; g ≈ 2.06 after UV irradiation and 2.04 before UV irradiation)—the g position of approximately 2.04–2.06 and the very large linewidth are characteristic of Cu^2+^ (d^9^) complexes with axial anisotropy, for which g ≈ 2.05–2.08 is typically observed. The hyperfine structure (A∥ ≈ 160–200 G) may remain unresolved due to geometric dispersion, spin exchange, or saturation, resulting in a broad, overlapping signal [47,48]. The increase in signal intensity after UV irradiation indicates a change in the EPR spectral response of Cu^2+^ centres. Phenoxyl radicals associated with curcuminoids, including demethoxycurcumin (DMC) and bisdemethoxycurcumin (BDMC), typically exhibit signals at g ≈ 2.003–2.006 [25,49,50,51]. Such a signal was not resolved in the present spectrum, possibly due to overlap with the broad Cu^2+^ component. The observed colour change may therefore be associated with photo-oxidative transformations of curcuminoids, potentially influenced by metal ions.

#### 2.1.3. EPR Study of the Epoxy Coating Formulation Containing Madder

**Figure 4:**
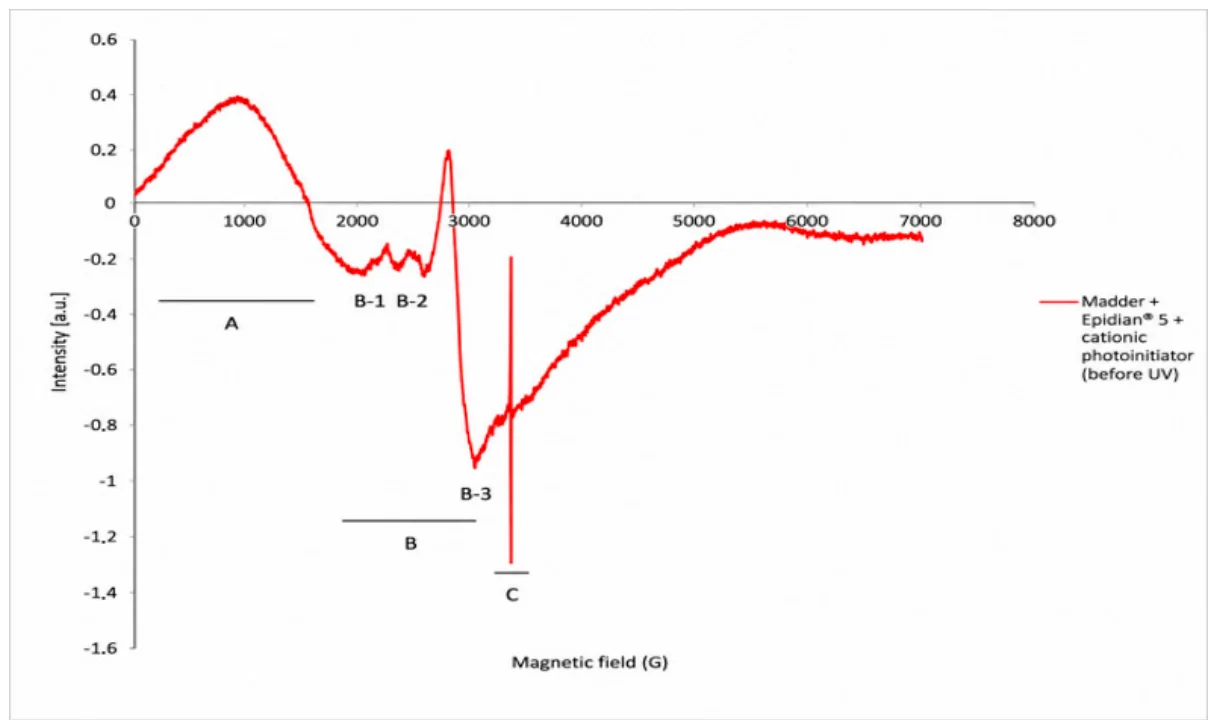
EPR spectrum of the mixture composed of madder, Epidian^®^ 5, and a cationic photoinitiator.

The prepared components were thoroughly mixed and placed in a quartz capillary, and then subjected to controlled UV irradiation. EPR spectra were recorded for a series of irradiation times: 0.5 min, 1 min, 2 min, 3 min, 4 min, 5 min, 6 min, 8 min, and 10 min (Figure 4, Table 3). The EPR spectrum shown in Figure 4 and the parameters listed in Table 3 refer to the epoxy coating formulation containing madder, Epidian^®^ 5 epoxy resin, and a cationic photoinitiator before UV irradiation.

Three main signal regions (A–C) were identified in the spectrum of the sample, corresponding to ferromagnetic interactions, a complex signal of Cu^2+^ ions, and stable organic radicals.

**Line A**—broad ferromagnetic component: in the low magnetic field region (approximately 1000–1800 G), a very broad band is observed, whose shape, lack of a distinct maximum, and nonlinear profile indicate ferromagnetic interactions characteristic of materials containing magnetic agglomerates or domains with a non-uniform field distribution. This signal is therefore macroscopic in nature rather than originating from a single paramagnetic ion. In the present sample, the large linewidth (Bpp > 1 kG) suggests that ferromagnetic interactions dominate in region A, rather than a classical Fe^3+^ signal.

**Line B**—broad Cu^2+^ signal with a visible hyperfine structure: the central part of the spectrum (approximately 2200–3200 G) exhibits a shape characteristic of Cu^2+^ ions (d^9^ system) in an anisotropic, heterogeneous ligand environment. Despite the broadening and partial overlap of the signals, a distinct hyperfine structure of the parallel component is observed in the spectrum, which is typical of the interaction between the unpaired electron and the copper nucleus (I = 3/2). On this basis, the approximate value of the hyperfine coupling parameter A∥ was estimated to be about 253 G, as determined from the spacing between adjacent hyperfine lines of the parallel component. The obtained result indicates the presence of Cu^2+^ centres in the investigated system and supports the interpretation of the signal as originating from Cu^2+^ ions in a distorted coordination environment. Owing to the linewidth and the incomplete resolution of all components, the analysis was partly qualitative [52,53].

**Line C**—in the region of g ≈ 2.001, a narrow line with a linewidth (Bpp) of a few gausses is observed, which is characteristic of semiquinone and phenoxyl radicals. The g value, close to that of the free electron (2.0023), together with the small linewidth, clearly indicates radicals present in aromatic systems, including the anthraquinone structures of plant-derived colorants [25,26]. The presence of this signal even before UV irradiation confirms the existence of a stable radical fraction in the sample [25].

**Figure 5:**
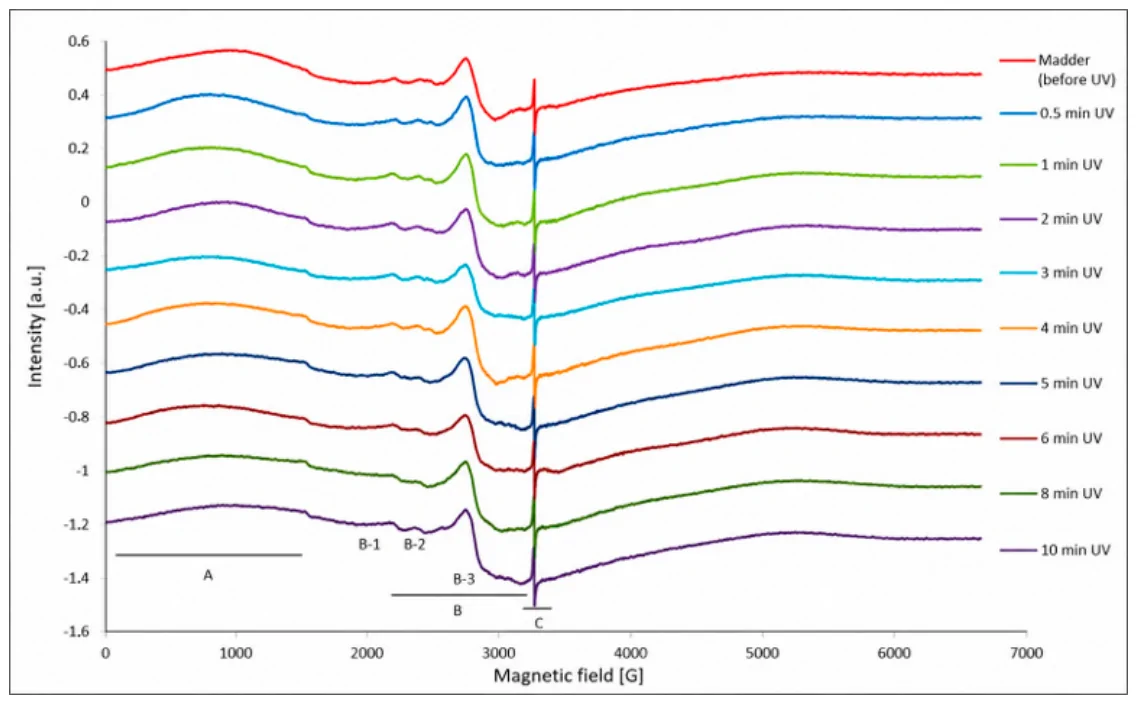
EPR spectra of the mixture containing madder extract, Epidian^®^ 5 epoxy resin, and the cationic photoinitiator triarylsulfonium hexafluoroantimonate, recorded after successive UV irradiation times (0–10 min).

The mixture (madder + Epidian 5 + cationic photoinitiator) was then subjected to controlled UV irradiation, and the resulting EPR spectra are presented in Figure 5 and Table 4. The spectrum recorded before UV irradiation (Figure 4; Table 3) was taken as the reference point.

**Line A** is characterized by a very large linewidth, Bpp (1.1–2.0 kG), and its intensity changes nonlinearly from 0.57 (before UV irradiation) to 0.21 (0.5 min), 1.21 (4 min, maximum), and 0.23 (10 min). Earlier interpretations associated this region with a signal originating from high-spin Fe^3+^ in a strongly distorted ligand environment, which would be consistent with the literature on the presence of Fe^3+^ in polymer matrices [24], as well as with the classical description of zero-field splitting (ZFS) parameters (D, E) used to characterize ligand-field asymmetry [1,47,54], in agreement with reports for Fe^3+^ in amorphous environments [55]. However, the character of the signal—its extremely large linewidth, strong dispersive component, and absence of hyperfine structure—clearly indicates that the dominant nature of this component is a broad ferromagnetic band, the intensity of which reflects the reorganization of magnetic interactions occurring during UV exposure. The reported peak-to-peak amplitudes describe changes in the EPR spectral profile and should not be interpreted as a direct quantitative measure of the amount of magnetic centres.

**Line B** covers the central part of the spectrum (approximately 2200–3200 G) and corresponds to a broad signal of Cu^2+^ ions (d^9^ system) in an anisotropic, heterogeneous ligand environment. In the series of spectra recorded after successive UV irradiation times, line B remains the dominant Cu^2+^ signal region; however, owing to line broadening and partial signal overlap, the resolution of the hyperfine structure deteriorates. For this reason, the analysis of time-dependent changes was based primarily on the descriptive parameters of line B, namely signal intensity, resonance position, and linewidth, whereas the labels B-1, B-2, and B-3 were treated as auxiliary analytical points used to organize the tabulated data. The intensities of points B-1 and B-2 remain low and variable, while B-3 constitutes the dominant analytical point within line B and reaches its maximum intensity after 4 min of UV irradiation. The B-1, B-2, and B-3 points are used solely to describe changes in the spectral profile and organize the tabulated data; their amplitudes should not be interpreted as a direct measure of Cu^2+^ concentration or chemical reaction kinetics. The observed changes in Bpp and intensity may indicate a rearrangement of the local environment of the Cu^2+^ centres and/or a change in the degree of signal overlap during UV exposure. Since full spin-Hamiltonian fitting (g∥, g⊥, A∥, A⊥) was not performed in this study, the interpretation in this respect should be regarded as partly qualitative [1,49,52].

**Line C** is a narrow signal with g ≈ 2.001 and Bpp ≈ 5.7–7.2 G, corresponding to stable semiquinone and phenoxyl radicals [25,26]. This is the most unambiguous and stable component of the spectrum. Its intensity increases quasi-monotonically from 0.861 (before UV irradiation) to 1.136 (after 10 min), indicating the gradual accumulation of stable aromatic radicals. At the same time, the increasing linewidth points to growing dispersion of the radical microenvironments and progressive changes in the anthraquinone chromophores of madder [25,26].

Overall, the results indicate that the analysed system behaves as a coupled photoredox system: the broad component A reflects the reorganization of magnetic interactions, component B documents the dynamic redox and coordination processes of Cu^2+^ ions [1], whereas component C corresponds to the formation and accumulation of stable semiquinone radicals directly responsible for the colour change. The broad metal-related bands may temporarily mask signals from short-lived species; however, the monotonic increase in component C provides clear evidence that the formation of madder chromophore radicals is the key mechanism responsible for colour change during UV exposure [25,26].

#### 2.1.4. EPR Study of the Epoxy Coating Formulation Containing Turmeric

**Figure 6:**
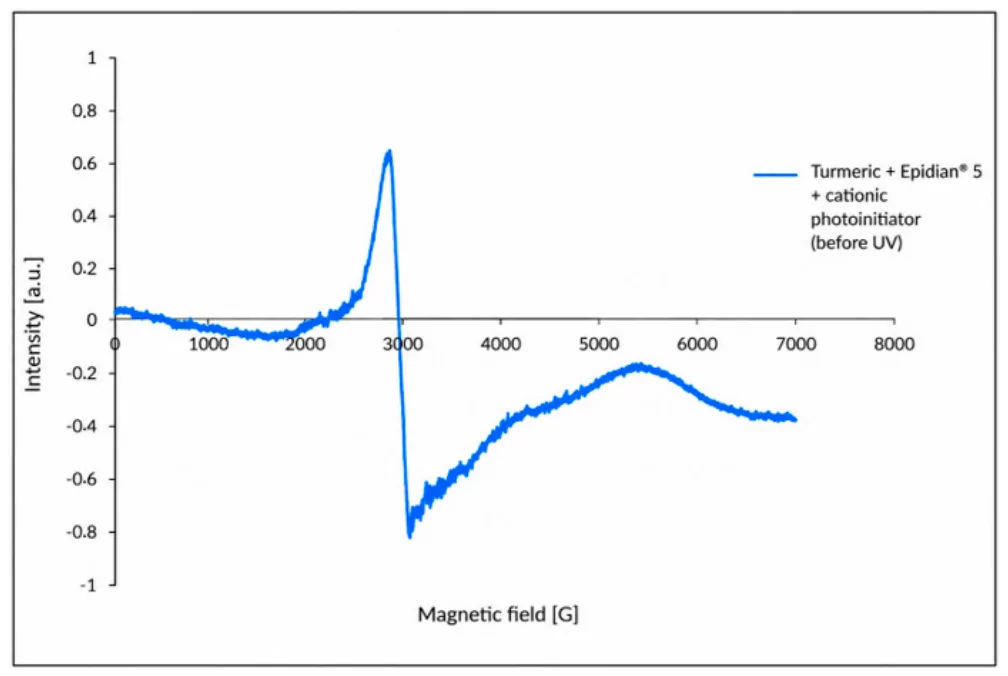
EPR spectrum of the mixture containing turmeric, Epidian^®^ 5, and a cationic photoinitiator.

Figure 6 and Table 5 present the EPR spectrum of the coating formulation containing turmeric, Epidian^®^ 5, and a cationic photoinitiator, recorded before exposure to UV radiation. In contrast to the madder-based systems, the spectrum exhibits a different character, being dominated by a broad line in the region of g ≈ 2.26 (B_0_ ≈ 2983 G, Bpp ≈ 214 G, I = 0.9374 a.u.).

The EPR spectrum of the coating formulation containing turmeric, Epidian^®^ 5, and a cationic photoinitiator before UV irradiation (Figure 6, Table 5) exhibits a single broad signal with parameters of g = 2.26 and Bpp ≈ 214 G. The broad signal at g ≈ 2.26 may be associated with transition-metal-related paramagnetic centres. These parameters are consistent with a signal originating from Cu^2+^ ions in an asymmetric environment, with typical values of g∥ ~ 2.2–2.3 and g⊥ ~ 2.05 [1]. The perpendicular component of Cu^2+^ centres (g⊥ ≈ 2.02–2.15) lies close to the spectral region of organic radicals, typically observed near g ≈ 2.00, which may result in partial spectral overlap. Furthermore, the observed signal at g ≈ 2.26 falls within the typical range of the parallel component of Cu^2+^ centres (g∥ ≈ 2.20–2.45). Comparison of the EPR spectra recorded before UV irradiation indicates that adding the photoinitiator to powdered turmeric does not substantially alter its spectral profile. Similarly, combining turmeric with Epidian^®^ 5 resin in the absence of the photoinitiator does not produce a comparable change, while the neat resin exhibits no detectable EPR signal under the applied measurement conditions. In contrast, the three-component formulation containing turmeric, the cationic photoinitiator, and Epidian^®^ 5 exhibits a modified spectral profile, including a shift in the resonance position. These observations suggest that interactions between the formulation components may modify the local environment of pre-existing paramagnetic centres. A possible explanation involves changes in the coordination environment of trace transition-metal ions originating from the natural colorant or photoinitiator. Functional groups of the epoxy resin, including epoxy and hydroxyl groups in the presence of the photoinitiator, may interact with these ions and modify their local ligand environment. Such interactions may influence the coordination geometry, g-tensor anisotropy, resonance positions, and linewidths. Therefore, the observed spectral changes do not necessarily indicate the formation of new paramagnetic centres, but may reflect changes in the EPR response of pre-existing metal-ion centres.

The presence of this signal may be associated with transition-metal ions introduced with the photoinitiator, since sulfonium hexafluoroantimonate salts may contain trace metal impurities, or with ions present in the curcuminoid colorant itself, which is known to form stable complexes with copper and other metal ions [50]. However, the unambiguous assignment of the broad signal at g ≈ 2.26 to Cu^2+^ centres remains challenging because of spectral broadening and the possible overlap of contributions from different paramagnetic species. Although its position and broadening are consistent with Cu^2+^ centres, the absence of clearly resolved hyperfine splitting and the possible overlap with organic radical signals prevent an unambiguous separation of these contributions. Therefore, the individual contributions of Cu^2+^ species and organic radicals cannot be quantitatively distinguished based on the recorded EPR spectra alone.

**Figure 7:**
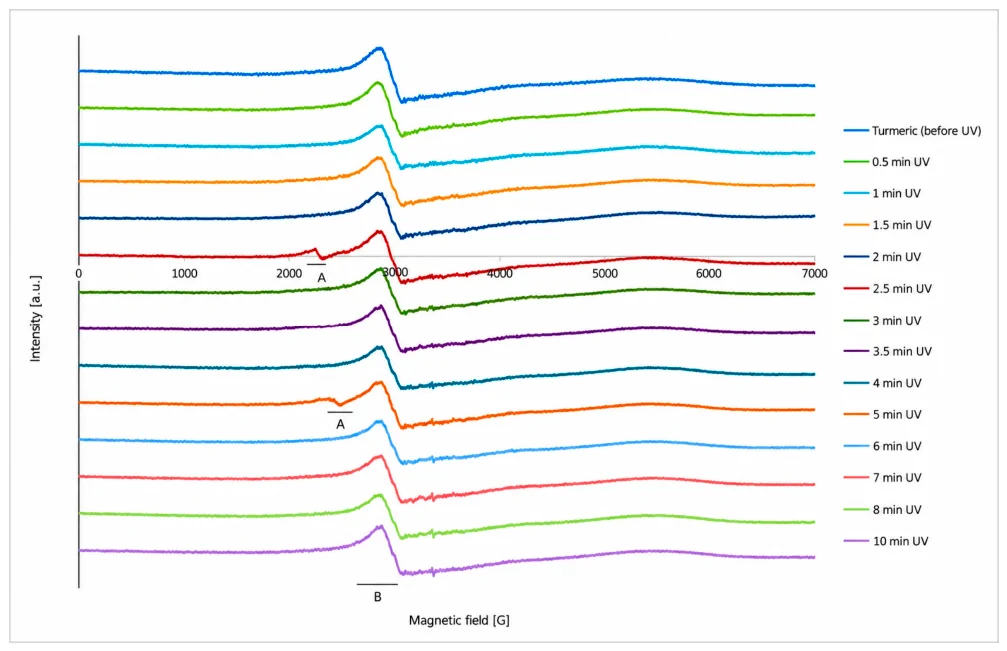
EPR spectra of the mixture containing turmeric, Epidian^®^ 5, and a cationic photoinitiator, recorded after UV irradiation for 0.5, 1, 2, 3, 4, 5, 6, 8, and 10 min.

After 0.5 min of UV irradiation (Figure 7, Table 5), the amplitude of the broad EPR signal was 0.9339 a.u., compared with 0.9374 a.u. before irradiation, while the g-factor remained close to 2.26–2.27. After 1 min, the amplitude decreased to 0.6886 a.u. Previous studies have described radical-related reactions of curcuminoids, including curcumin and its derivatives [50,51], as well as UV-induced changes in curcumin stability [56]. However, the observed EPR signal cannot be directly attributed to phenoxyl radicals based solely on its resonance position.

After approximately 2.5–5 min of irradiation, an additional narrow signal appears at g ≈ 2.95 (B_0_ ≈ 2287–2450 G), which may be assigned to Fe^3+^ ions S = 1/2 [1,57,58,59,60]. This suggests that, during photodegradation of the system, trace metal impurities become activated and act as redox centres catalysing radical transformations. At the same time, fluctuations in the intensity of the main signal in the region of g ≈ 2.26 are observed.

After 5 min, an additional spectral component at g ≈ 2.75 is observed alongside the persistent broad signal at g ≈ 2.26. In contrast, EPR signals of phenoxyl radicals reported in plant-derived and radical–copper systems occur close to g ≈ 2.00 [25,26]. Therefore, these signals cannot be unambiguously assigned to phenoxyl radicals based solely on their g-values. The analysis of the turmeric + Epidian^®^ 5 + cationic photoinitiator system indicates complex changes in the EPR spectral profile during UV irradiation, including variations in signal intensity and linewidth and the appearance of additional paramagnetic components, suggesting changes in the local environment and interactions of paramagnetic centres.

#### 2.1.5. EPR Study of the Polyester Coating Formulation Containing Madder

**Figure 8:**
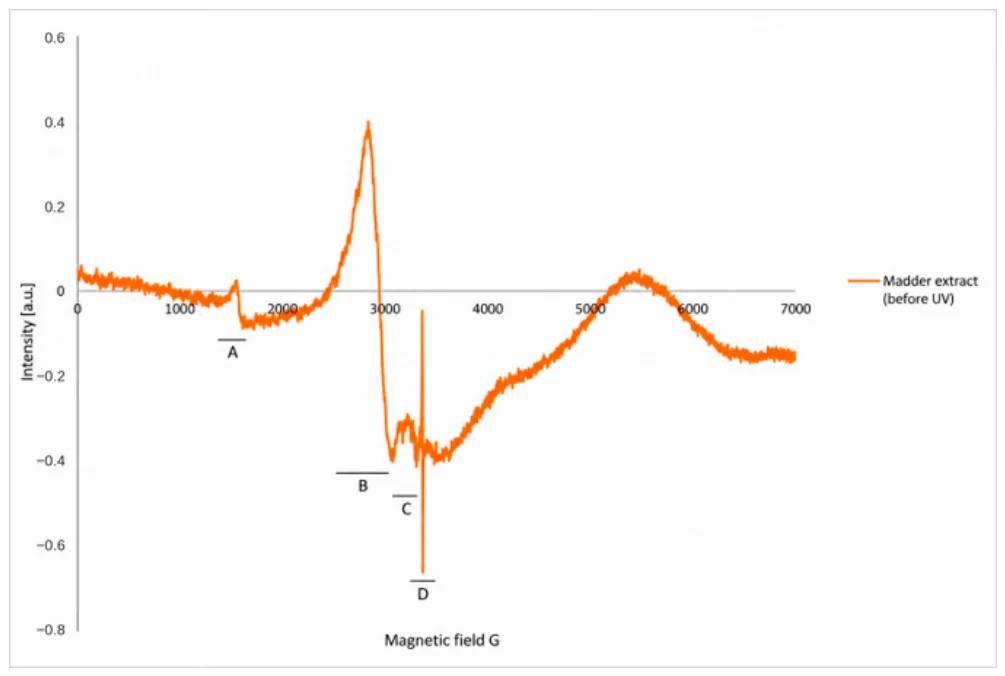
EPR spectrum of the coating formulation containing madder extract (Rubia tinctorum), the photoinitiator Irgacure 651, and Polimal^®^ 109 resin, before UV irradiation.

In the next part of the experiment, a coating formulation containing a natural colorant, a photoinitiator, and a photocurable resin was analysed. The formulation contained madder extract (Rubia tinctorum, 0.5 g), used as the colorant (madder red dye extract), the radical photoinitiator Irgacure 651 (0.02 g), and Polimal^®^ 109 unsaturated polyester resin (9.31 g) as the polymer matrix. The mixture was placed in a capillary tube and then subjected to controlled UV irradiation. The EPR spectrum recorded for the non-irradiated sample is shown in Figure 8. It serves as the reference point for the subsequent experiments, in which the samples were subjected to controlled UV exposure.

Based on this spectrum, four characteristic regions (A–D) were identified and subsequently analysed quantitatively (Table 6).

Based on the spectrum (Figure 8, Table 6), the presence of three main classes of signals was identified: high-spin Fe^3+^ (region A), a broad Cu^2+^ (d^9^) signal in the range of g ≈ 2.05–2.3 (regions B–C), and a narrow signal attributed to organic radicals at g ≈ 2.001 (region D). This spectrum was taken as the reference point for further comparative analyses. The Fe^3+^ signal (S = 5/2) at B_0_ ≈ 1585 G (g ≈ 4.26; Bpp ≈ 59 G; line A) corresponds to Fe^3+^ in a rhombic environment [24]. The dominant signal is a broad line at g ≈ 2.28 (B_0_ ≈ 2961 G; Bpp ≈ 202 G; line B), indicating the presence of Cu^2+^ [47]. In addition to these signals, a narrow line is recorded at g ≈ 2.001 (B_0_ ≈ 3374 G; Bpp ≈ 6.0 G; line D), corresponding to organic radicals of the phenoxyl or semiquinone type. The g and Bpp values are consistent with literature data for phenoxyl radicals and o- or p-semiquinones in aromatic compounds (g ~ 2.003–2.006) [25,26].

**Figure 9:**
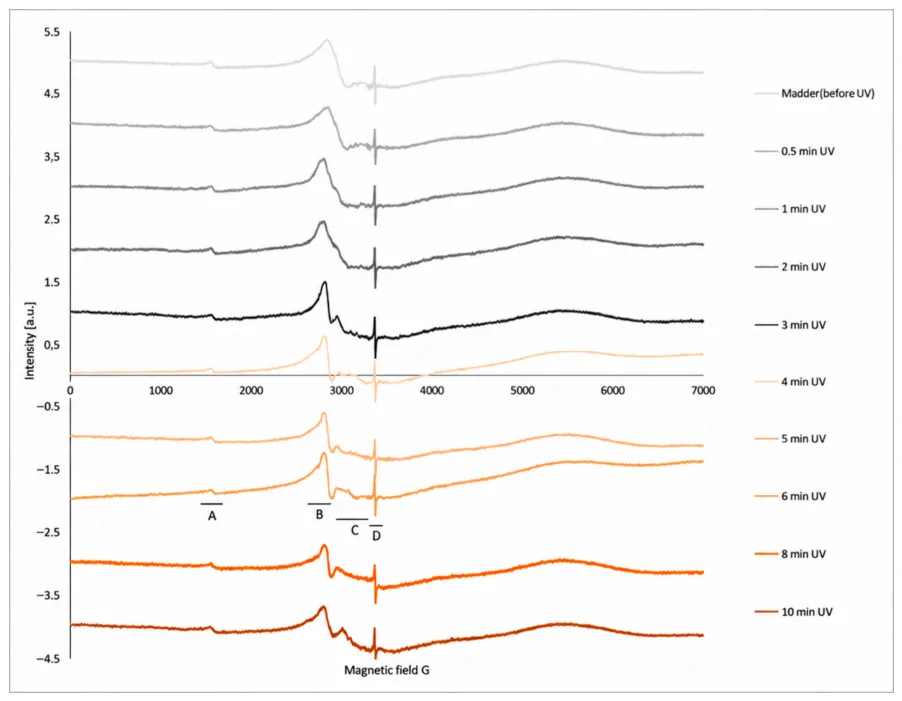
EPR spectra of the coating formulation containing madder extract (Rubia tinctorum), the photoinitiator Irgacure 651, and Polimal^®^ 109 resin, recorded after UV irradiation for 0.5, 1, 2, 3, 4, 5, 6, and 8 min.

In the subsequent stages of the study, a systematic analysis of changes in the intensity and shape of the EPR lines was carried out after successive UV irradiation times (0.5–10 min). Particular attention was paid to the evolution of signals in regions A–D, comprising signals originating from metal ions (Fe^3+^, Cu^2+^) and organic radicals. The obtained spectra (Figure 9) made it possible to observe the dynamics of the formation and decay of individual paramagnetic centres and thus to follow the mechanism of colour change in the coating over time. The EPR signal parameters obtained after UV irradiation of the coating formulation containing madder, Polimal^®^ 109, and Irgacure 651 at 0.5, 1, 2, 3, 4, 5, 6, 8, and 10 min were analysed (Table 7).

**Line A**—the signal at g ≈ 4.25 (B_0_ ≈ 1585–1586 G, Bpp ≈ 65 G) corresponds to classical Fe^3+^ (S = 5/2) in a rhombic environment. Its only slight changes in intensity (0.0359–0.055 a.u.) indicate that the amount of Fe^3+^ centres remains relatively constant throughout the entire UV irradiation process and does not directly determine the colour change [24].

**Line B**—after 0.5–2 min of irradiation, the intensity of line B decreases to approximately 0.29 a.u., which suggests partial reduction of Cu^2+^ or a change in its coordination geometry [1,48]. In the third minute, the signal changes shape: an additional, broader component appears at the base of the signal, indicating splitting into two populations:

B-1: g ≈ 2.36; B_0_ ≈ 2861 G; Bpp ≈ 108 G; I ≈ 0.037 a.u.—a broader component, probably associated with modified Cu^2+^ coordination and stronger spin–spin interactions.

B-2: g ≈ 2.36; B_0_ ≈ 2857 G; Bpp ≈ 77.6 G; I ≈ 0.0477 a.u.—corresponding to Cu^2+^ centres with a geometry close to tetragonal.

After 3–6 min of irradiation, the total intensity increases (0.60–0.61 a.u.), and after 8–10 min it stabilizes at the level of 0.30–0.40 a.u. Changes in Bpp and shifts in g indicate a dynamic equilibrium between Cu^+^ and Cu^2+^, as well as photoinduced processes of local-environment reorganization [1,25,51,52]. Line B therefore reflects the active involvement of Cu^2+^ in photoredox processes, with the possible coexistence of other metallic centres typical of natural colorants.

**Line C** (g ≈ 2.02–2.26) exhibits pronounced changes in its spectral profile during UV irradiation. After 0.5 min, the signal amplitude decreases from 0.1085 to 0.00779 a.u., while Bpp decreases from 212 to approximately 169 G. At 1–2 min, the line becomes narrower (Bpp ≈ 30–70 G), with apparent g-values of 2.06–2.08. At 3 min, the amplitude reaches approximately 0.0681 a.u., accompanied by a shift in g towards 2.25. At 4, 5, and 6 min, the recorded amplitudes are 0.0975, 0.00351, and 0.1225 a.u., respectively, while Bpp varies between 51.9 and 188 G. After 8–10 min, the amplitudes range from 0.0058 to 0.0373 a.u., with Bpp ≈ 176–196 G.

These changes may reflect variations in the relative contributions and local environments of overlapping paramagnetic centres, potentially including Cu^2+^-related species [52,61,62,63,64,65]. High-spin Fe^3+^ ions do not exhibit EPR signals exclusively at g ≈ 4.3. Depending on the local symmetry, zero-field splitting, and coordination environment, high-spin Fe^3+^ centres may also exhibit EPR features near g ≈ 2.0 [57]. Furthermore, organic radical signals are not necessarily narrow in condensed or structurally heterogeneous systems, where line broadening and spectral overlap may occur. Nevertheless, the broad spectral features of Line C do not, by themselves, establish the simultaneous contribution of high-spin Fe^3+^ ions and organic radicals. Therefore, the interpretation of Line C is based on its experimentally observed spectral parameters and the possible overlap of different paramagnetic contributions, including transition-metal-related centres. A separate narrow component near g ≈ 2.001 (Line D) is consistent with an organic-radical-related response [25,66]. The reported amplitude variations were determined experimentally from the recorded EPR spectra. Since the linewidth and degree of spectral overlap also change during UV irradiation, these amplitudes should not be interpreted as direct quantitative measures of the concentrations of individual paramagnetic centres.

**Line D** (g ≈ 2.001; semiquinone/phenoxyl radicals) is the most stable and characteristic signal in the system. Its initial intensity (0.465 a.u.) increases to a maximum of approximately 0.51 a.u. after 10 min of UV irradiation, while Bpp increases from ~6.0 to ~7.4 G. These parameters (g ≈ 2.001 and a linewidth of a few gauss) correspond to semiquinone and phenoxyl radicals formed as a result of irradiation of anthraquinone-type compounds present in madder [25,26]. The accumulation of these stable radicals is crucial for the macroscopically observed colour change, which results from π-electron delocalization within the system of conjugated double bonds and from local disruption of π-conjugation (Figure 9), ultimately leading to a shift in the absorption bands and hence to a change in colour.

In madder-containing formulations, UV-induced colour changes may be associated with radical reactions involving anthraquinone chromophores, potentially influenced by trace transition-metal centres.

Taken together, the EPR results show that the analysed formulations cannot be described by a single paramagnetic process. The stable organic-radical signals provide the clearest evidence of progressive changes in the natural colorants, whereas the broad and partially overlapping signals attributed to transition-metal centres show stronger temporal fluctuations and, in several cases, allow only partly qualitative interpretation. Accordingly, the assignment of individual broad components should be treated with caution where hyperfine structure is not fully resolved or where several paramagnetic centres may contribute simultaneously. This limitation is important when relating the EPR response directly to the observed colour change and is the reason why the EPR results were considered together with FTIR spectroscopy and colour measurements.

### 2.2. FTIR Analysis

The aim of the FTIR analysis was to verify whether the changes observed by EPR—namely the growth and stabilization of paramagnetic/radical centres and the role of redox components—are confirmed by an independent spectroscopic technique, and whether a common, stepwise transformation mechanism leading to colour change can be identified for both colorants.

#### 2.2.1. FTIR Analysis of the Epoxy Coating Formulation Containing Madder

**Figure 10:**
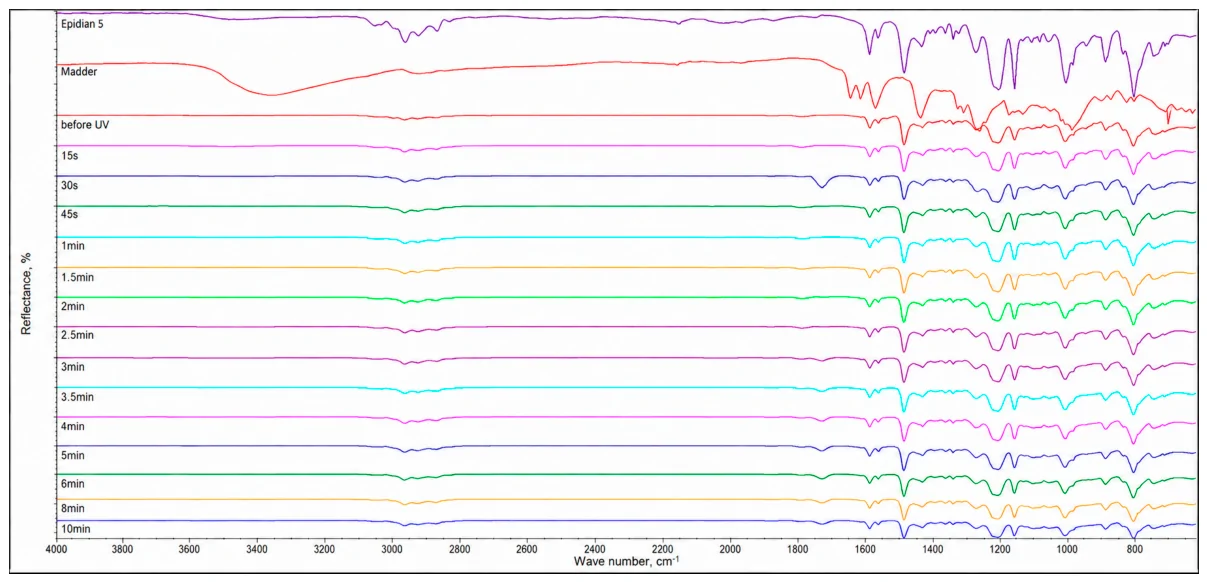
Comparison of the FTIR spectra of epoxy resin Epidian^®^ 5, madder and of the coating formulation containing madder and cationic photoinitiator triarylsulfonium hexafluoroantimonate, before and after UV irradiation.

The FTIR spectra of epoxy resin Epidian^®^ 5, madder and of the epoxy coating formulation containing madder, recorded before and after UV irradiation, are presented in Figure 10.

The FTIR spectra of the epoxy coating formulation containing madder indicate that several processes occur simultaneously under UV irradiation. During UV exposure, effects associated with resin photocuring can be observed: the band corresponding to O–H stretching vibrations in the range 3200–3600 cm^−1^ increases in intensity and becomes broader, while in the range 1250–1000 cm^−1^ an increase in the intensity of the asymmetric stretching bands characteristic of ether C–O–C groups is observed. Moreover, a decrease in the intensity of the band attributed to the epoxy ring vibration at approximately 915 cm^−1^ indicates opening of the epoxy ring.

**Figure 11:**
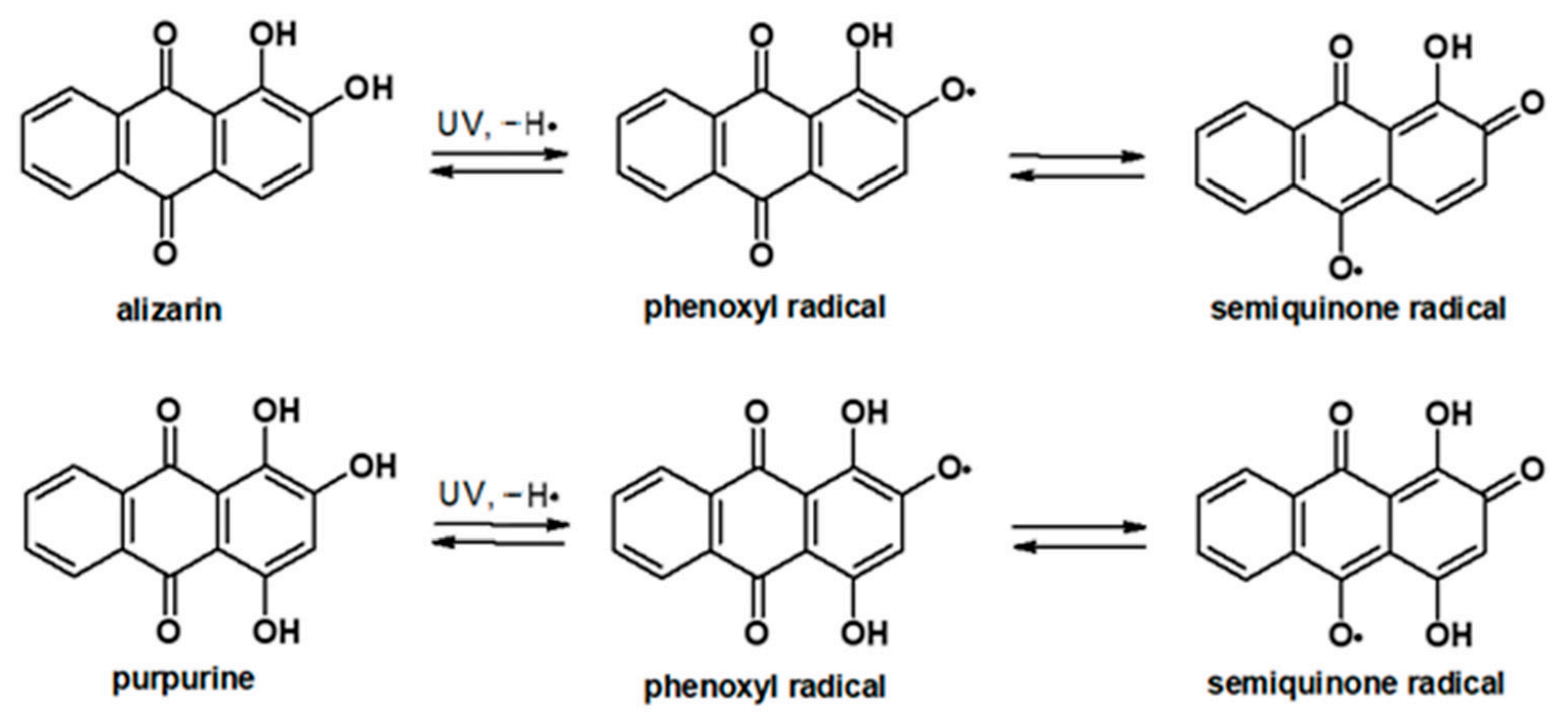
Proposed reaction pathways involving phenoxyl and semiquinone radicals in alizarin and purpurin during UV irradiation.

The bands at 1650 and 1675 cm^−1^ come from carbonyl groups in the anthraquinone rings of madder. These bands are poorly visible in the coating formulation due to the low madder content. After 30 s of exposure, a new band appears at approximately 1750 cm^−1^, which then disappears during further exposure, after which its intensity increases again. attributed to vibrations of carbonyl groups, probably formed as a result of radical transformations. then fades away with further exposure before increasing in intensity again. The experimentally observed changes in the FTIR absorption bands, particularly in the carbonyl region, indicate UV-induced structural transformations of the coating formulation. In parallel, the EPR measurements revealed changes in the paramagnetic response during irradiation. The correlation between these observations is consistent with possible radical reactions involving the anthraquinone structures of alizarin and purpurin. On this basis, the formation of phenoxyl and semiquinone radicals and their possible interconversion are proposed as plausible reaction pathways. However, the exact radical structures, their positions within the anthraquinone rings, and the individual reaction steps were not directly identified experimentally. Therefore, the reaction pathways presented in Figure 11 should be regarded as proposed mechanistic interpretations rather than experimentally confirmed transformations [67].

#### 2.2.2. FTIR Analysis of the Epoxy Coating Formulation Containing Turmeric

**Figure 12:**
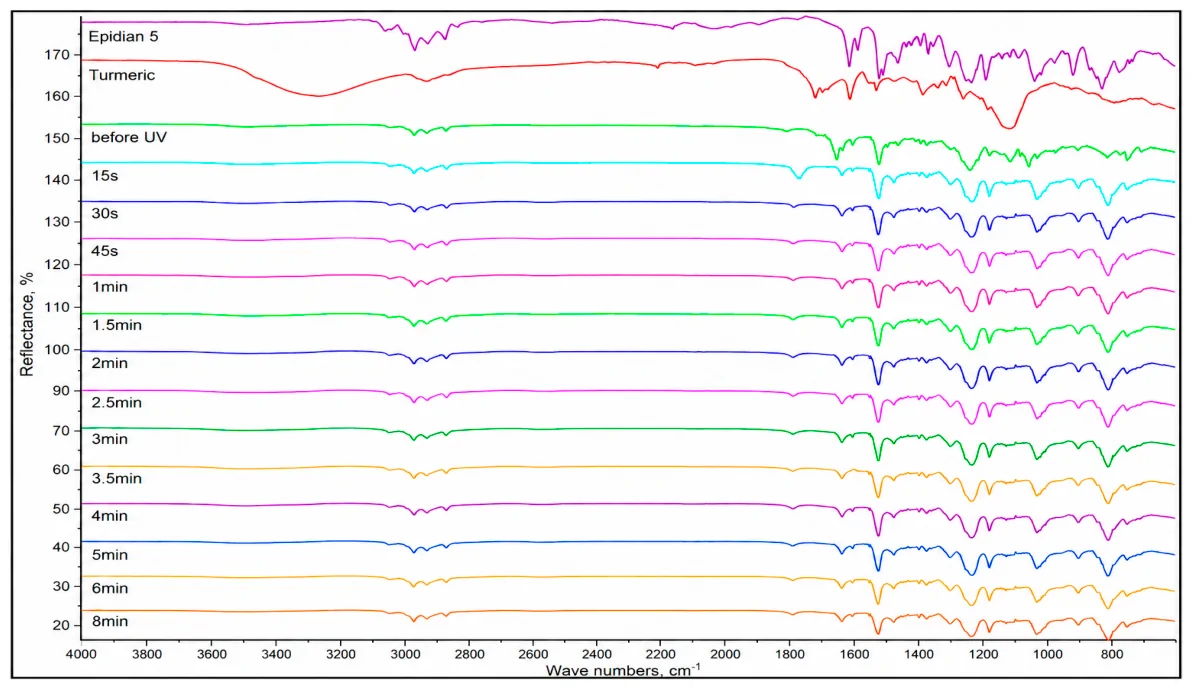
Comparison of the FTIR spectra of epoxy resin Epidian^®^ 5, turmeric and of the epoxy coating formulation containing turmeric and cationic photoinitiator triarylsulfonium hexafluoroantimonate, before and after UV irradiation.

The FTIR spectra of epoxy resin Epidian^®^ 5, turmeric and of the epoxy coating formulation containing turmeric, recorded before and after UV irradiation, are presented in Figure 12.

The FTIR spectra of the epoxy coating formulation containing turmeric indicate a course of changes under UV irradiation similar to that observed for the mixtures containing madder. Throughout the entire investigated time range, processes associated with resin photocuring take place: the band corresponding to O–H stretching vibrations in the range 3200–3600 cm^−1^ increases in intensity and becomes broader; in the range 1250–1000 cm^−1^, an increase in the intensity of the asymmetric stretching bands characteristic of ether C–O–C groups is observed; and at approximately 915 cm^−1^, a decrease in the intensity of the band attributed to epoxy ring vibrations is noted.

The band at approximately 1700 cm^−1^ originates from the carbonyl groups of curcumin. Similarly to the mixture containing madder, a band appears at approximately 1780 cm^−1^ after 30 s of irradiation, which is assigned to the vibrations of carbonyl groups. During further irradiation, this band disappears and then increases in intensity once again. The variation in the intensity of this band under irradiation indicates the disappearance and re-formation of carbonyl groups.

**Figure 13:**
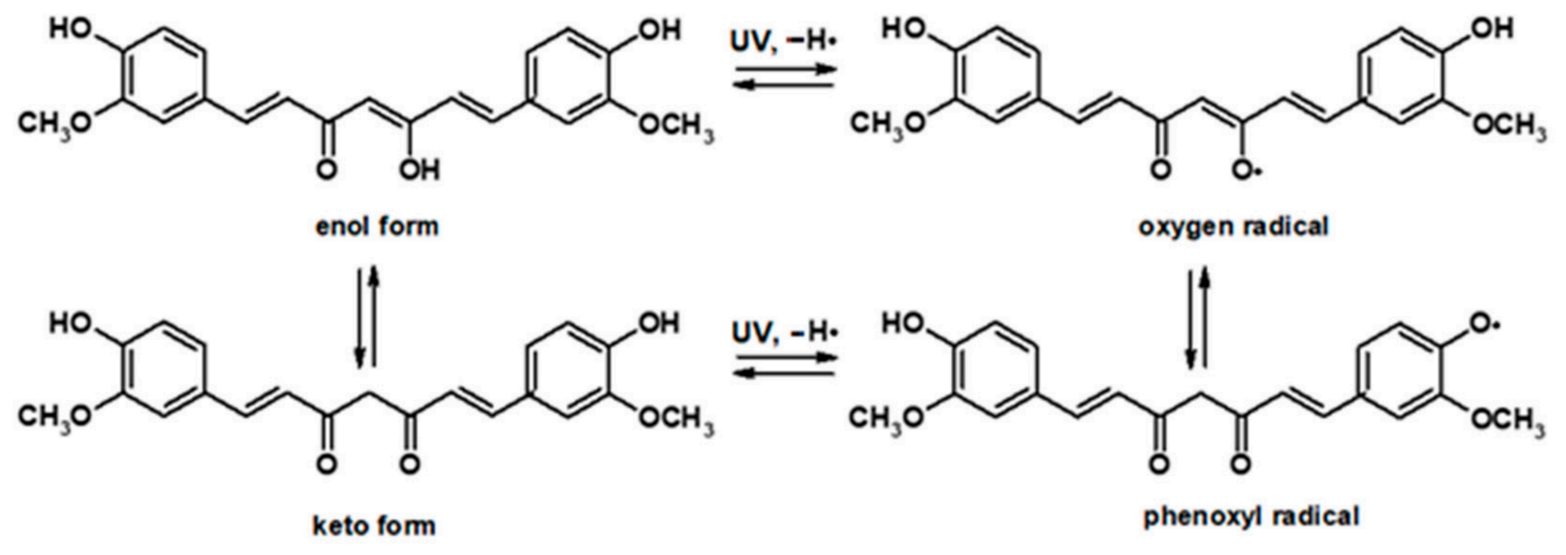
Proposed reaction pathways involving oxygen-centred and phenoxyl radicals in curcumin during UV irradiation.

The observed changes in the FTIR carbonyl absorption band indicate UV-induced structural transformations in the turmeric-containing coating formulation. The corresponding changes in the EPR response are consistent with the involvement of paramagnetic species during irradiation. Based on these combined observations, the possible formation of phenoxyl and oxygen-centred radical intermediates, accompanied by transformations involving the keto–enol forms of curcumin, is proposed as a plausible reaction pathway. However, the specific radical structures and individual reaction steps cannot be directly confirmed from the recorded FTIR and EPR spectra. Therefore, the mechanism illustrated in Figure 13 represents a proposed interpretation of the experimental observations rather than an unequivocally established reaction pathway.

#### 2.2.3. FTIR Analysis of the Polyester Coating Formulation Containing Madder

**Figure 14:**
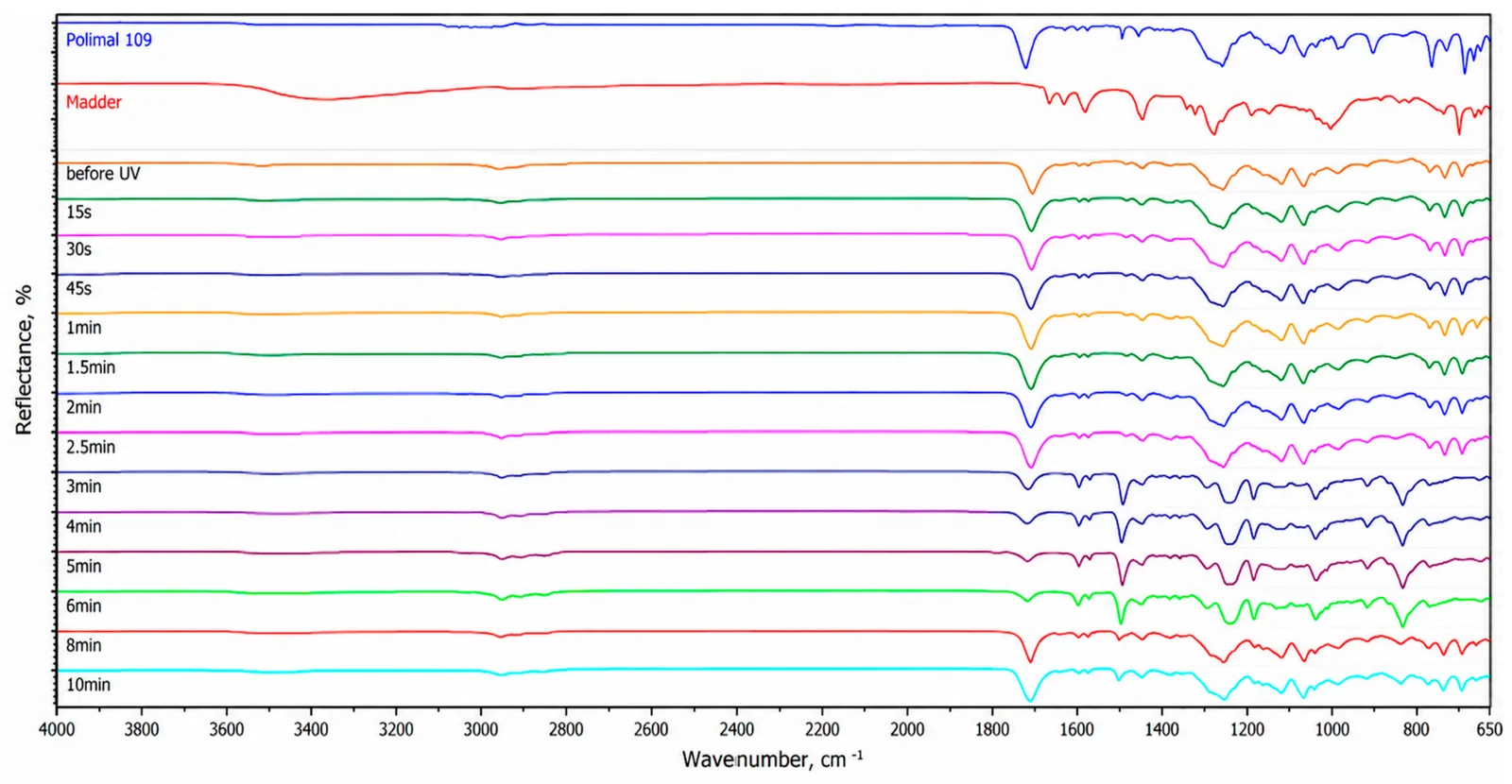
Comparison of the FTIR spectra of polyester resin Polimal^®^ 109, madder and of the polyester coating formulation containing madder and radical photoinitiator Irgacure 651, before and after UV irradiation.

The FTIR spectra of polyester resin Polimal^®^ 109, madder and of the polyester coating formulation containing madder, recorded before and after UV irradiation, are presented in Figure 14.

The FTIR spectra of the polyester coating formulations containing madder indicate that the changes induced in its chemical structure by UV irradiation proceed more slowly than in the mixture containing the epoxy resin. Only after 3 min of irradiation, changes become apparent in FTIR spectra: the intensity of the bands attributed to the carbonyl group vibrations of the resin at approximately 1710–1735 cm^−1^ and the bands at 1650 cm^−1^ and 1675 cm^−1^ coming from carbonyl groups in the anthraquinone rings of madder decreases, as does the intensity of the C–O stretching vibrations at approximately 1280 cm^−1^, corresponding to OH groups attached to aromatic rings of madder and esters groups of polyester resin. At the same time, the intensity of the bands at 1590 cm^−1^, assigned to stretching vibrations of the double bonds in the anthraquinone rings of madder constituents, increases. This indicates changes in the structure of aromatic rings of madder during UV irradiation. In the case of the mixture containing madder and epoxy resin, the disappearance of the band at 1750 cm^−1^ was observed after only 45 s of exposure. Furthermore, no changes were observed at approximately 1620 cm^−1^ and 1510 cm^−1^.

In contrast to the mixture containing madder and the epoxy resin, the changes in the presence of the polyester resin occur more slowly, but after they start, in addition to the transformation of carbonyl groups into radicals, differences in aromatic structures are observed. The different responses of madder-containing epoxy and polyester formulations to UV irradiation may be associated not only with differences in the chemical structure of the polymer matrices but also with the photoinitiators and polymerization mechanisms employed. The epoxy formulation undergoes cationic polymerization initiated by triarylsulfonium hexafluoroantimonate, whereas the unsaturated polyester formulation undergoes free-radical polymerization initiated by Irgacure 651. These differences may influence the kinetics of curing and the photochemical environment of the colorant, thereby contributing to the observed differences in the evolution of the FTIR spectra during UV exposure [68,69].

The comparison of the FTIR results therefore indicates that the observed differences in the rate and character of UV-induced transformations may reflect the combined influence of the polymer matrix, photoinitiator, and polymerization mechanism, rather than the effect of the polymer matrix alone. For the epoxy coating formulations, changes associated with the colorants appear earlier, whereas in the polyester coating formulation containing madder they become apparent only after a longer irradiation time. The interpretation of individual bands must nevertheless take into account their overlap with bands originating from the polymer matrix, particularly in the carbonyl region. For this reason, the FTIR results are interpreted here in correlation with the EPR response rather than as independent proof of the radical mechanism.

### 2.3. Colour Measurements Results

#### 2.3.1. Results of Colour Measurements for the Epoxy Coating Formulation Containing Madder

**Figure 15:**
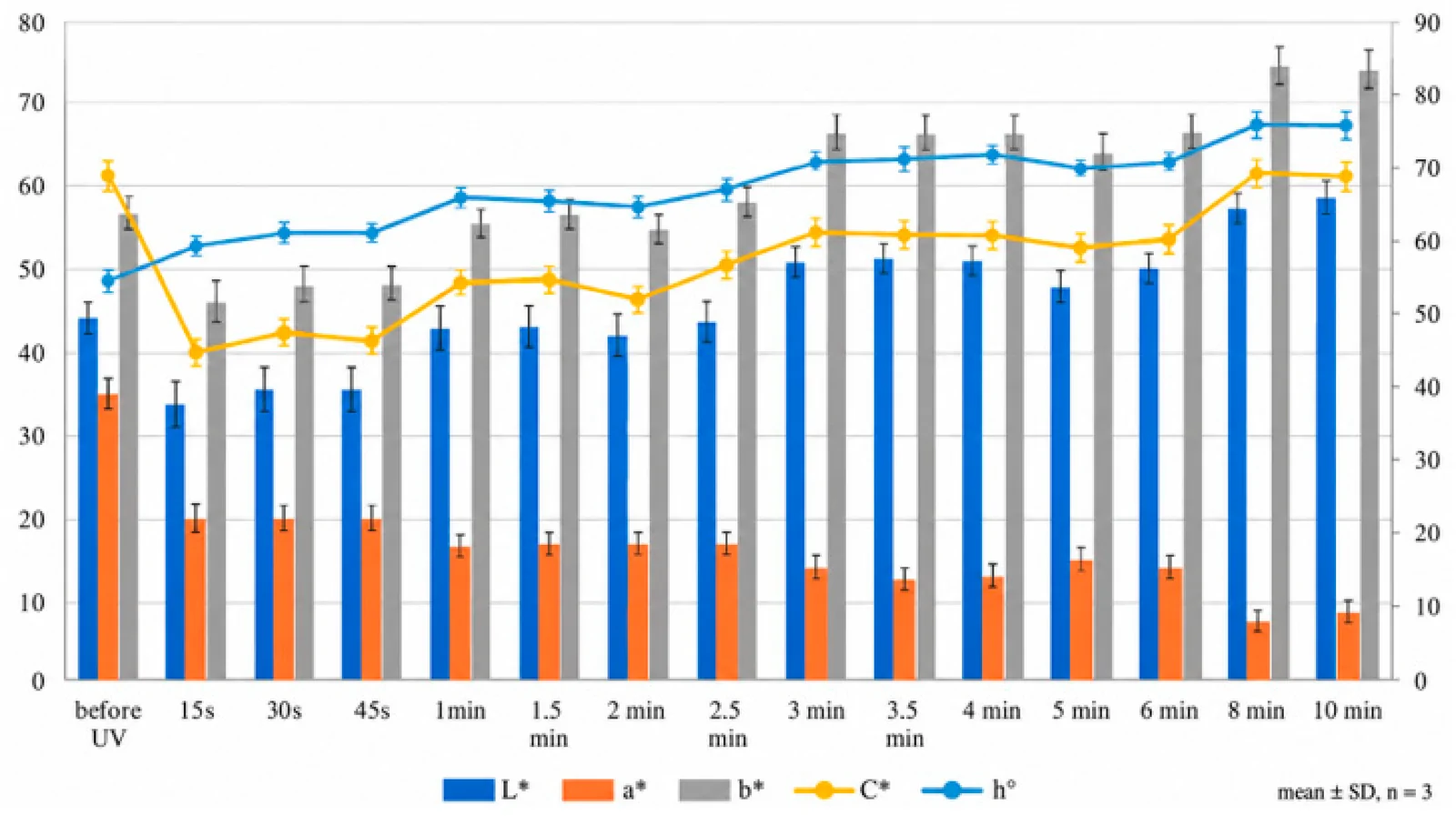
Changes in the colour coordinates in the CIELAB space (L*, a*, b*), together with the chroma (C*) and hue angle (h°), for the epoxy coating formulation containing madder during UVB exposure from 15 s to 10 min.

The results of the colour measurements for the epoxy coating formulation containing madder, recorded before and after UV irradiation, are presented in Figure 15.

The initial state (before UV exposure) was characterized by moderate lightness (L* = 44.7), a high positive a* value (34.93), and a high b* value (59.11), indicating an intense reddish-yellow colour. The high colour saturation (C* = 68.66) and hue angle (h° = 59.42°) confirm the dominance of a yellowish-orange tone.

Changes in lightness (L*)

Immediately after the onset of UVB exposure, a distinct decrease in lightness was observed. After 15 s, L* decreased from 44.7 to 33.81, indicating rapid darkening of the system. From 30 s up to 2.5 min of irradiation, the lightness gradually increased and stabilized in the range of 42–44, indicating a partial recovery of lightness following the initial darkening.

After 3 min, a further increase in L* was observed, reaching approximately 50–51 in the 3–4 min irradiation range. In the final stage of exposure (8–10 min), L* increased to 58.48–58.56, indicating a marked brightening compared with the initial values.

Changes along the red–green axis (a*)

Initially, the high a* value (34.93) indicated a more intense red colour. After 15 s, a sharp decrease in a* to 20.19 was observed, reflecting a reduction in red colour intensity. During UV exposure from 30 s to 4 min, the a* value gradually decreased to 12.67. In the final stage (8–10 min), the a* parameter dropped to 8.12–8.97, corresponding to approximately 25% of its initial value. This indicates a systematic loss of red colour during prolonged irradiation. The decreasing a* trend indicates the destruction of the chromophore structure of madder, while the partial recovery of lightness (L*) observed during the initial stages of irradiation does not necessarily indicate regeneration of the original madder chromophores but may be caused by processes occurring in the epoxy matrix.

Changes in the yellow component (b*)

The increase in b* from 59.11 to 83.83 during UV irradiation indicates a pronounced shift towards the yellow component, accompanied by a substantial decrease in a* from 34.93 to approximately 8–9. This behaviour may be attributed to the combined effects of photochemical transformations of anthraquinone colorants and photooxidative changes in the epoxy matrix. Previous studies have demonstrated that UV ageing of epoxy resins can lead to yellowing through the formation of new chromophoric oxidation products [68], while irradiation of alizarin can result in oxidative degradation and fading of the original chromophore [70]. Accordingly, the observed colour evolution is consistent with the possible coexistence of these processes. However, their relative contributions cannot be determined from the present color measurements alone.

Colour saturation (C*)

The C* parameter exhibited an initial decrease, from 68.66 to 48.73 after 15 s of irradiation, analogous to the decline observed for a* and b*. Between 1 and 4 min, a gradual increase in saturation was observed, reaching a level close to the initial value (67.49). In the final stage (8–10 min), the C* value increased to approximately 75.6, exceeding the initial value. An increase in the C* value may result from the photochemical yellowing of the epoxy matrix (b* = 83.83), combined with the fading of the madder (a* = 8.97).

*Changes in the hue angle (h°)*

The hue angle increased systematically from 59.42° (orange-red region) to 65–74° during the first minutes of exposure, and then to 78–79° after 3–6 min. In the final stage (8–10 min), the h° parameter reached 83.19, indicating a distinct shift toward the yellow color region. Such a significant change in the hue angle suggests that the visual color of the sample in the later stages of exposure is determined by the yellowing of the epoxy matrix and the fading of the madder.

#### 2.3.2. Results of Colour Measurements for the Epoxy Coating Formulation Containing Turmeric

**Figure 16:**
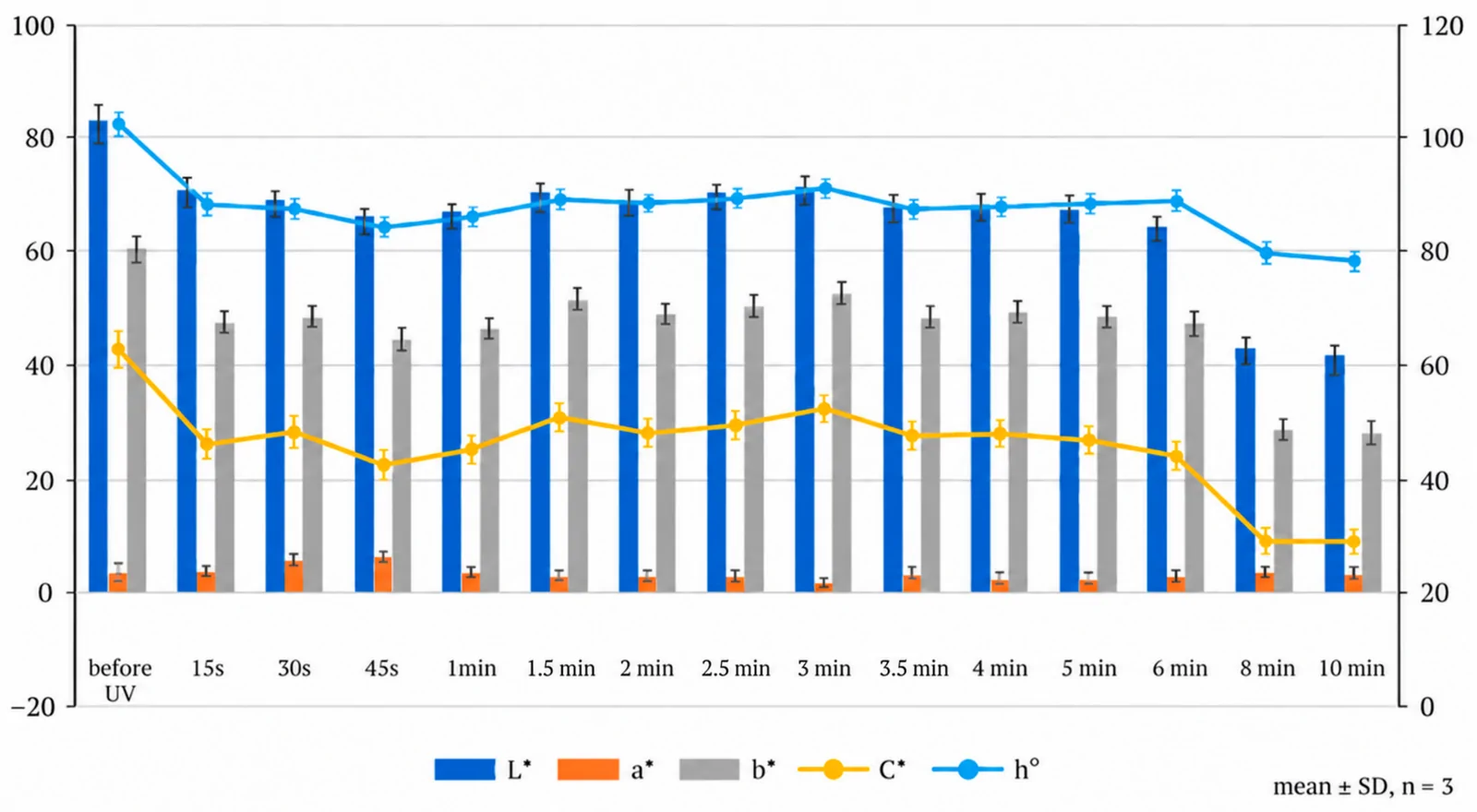
Changes in the colour coordinates in the CIELAB space (L*, a*, b*), together with the chroma (C*) and hue angle (h°), for the epoxy coating formulation containing turmeric during UVB exposure from 15 s to 10 min.

The results of the colour measurements for the epoxy coating formulation containing turmeric, recorded before and after UV irradiation, are presented in Figure 16.

The initial colour (before UV exposure) was characterized by high lightness (L* = 83.15) and a high value of the yellow component (b* = 60.57), together with a negative value of the a* parameter (a* = –10.04), indicating the dominance of the yellow component, while the negative a* value imparts a subtle greenish (lemon-like) tint. The initial colour saturation was high (C* = 61.40), while the hue angle was located in the yellow–green region (h° = 99.41°).

Changes in lightness (L*)

Immediately after the onset of UVB exposure, a sharp decrease in lightness was observed. After only 15 s, the L* value decreased from 83.15 to 69.47 (ΔL* ≈ –13.7), indicating rapid darkening of the sample. In the time interval from 15 s to 6 min, the lightness remained within a relatively narrow range (64.3–71.3). A distinct second phase of changes appeared during longer irradiation times. After 8–10 min, L* dropped sharply to 41.93–41.00, indicating strong darkening associated with structural changes in the colorant during UV irradiation.

Changes along the red–green axis (a*)

The most characteristic initial effect was a change in the sign of the a* parameter. After only 15 s, the a* value changed from –10.04 to +4.04, indicating a shift in colour from the greenish region toward red. In the interval 1.5–6 min, a* fluctuated around small positive values (~1.3–3.9), indicating partial stabilization of the red–green balance. After 8–10 min, a further increase in a* to 6.12–6.23 was observed, combined with a strong decrease in lightness, demonstrating the appearance of a brownish-olive colour.

Decrease in the yellow component (b*) and chroma (C*)

The initially high b* value (60.57) confirmed the intensely yellow colour of the sample. After 15 s of UVB exposure, the b* parameter decreased sharply to 45.06, indicating a rapid decline in yellow colour intensity. The minimum occurred after 45 s (b* = 41.79). In the interval 1.5–3 min, a partial increase in b* was observed (b* = 49.44–51.36), which may be related to the conversion of radicals into other forms (Figure 13). However, with prolonged irradiation, a distinct loss of yellowness occurred. After 8–10 min, b* decreased to 28.28–28.16, that is, to less than half of its initial value. The chroma parameter C* showed a trend analogous to that of b*, confirming the overall fading and desaturation of the system. C* decreased from 61.40 (before UV) to 45.24 (15 s), remained in the range of 44–51 up to 6 min, and then dropped sharply to about 28.9 after 8–10 min. The simultaneous decrease in b* and C* indicates progressive disruption of the system of conjugated π bonds responsible for the intense yellow colour, as well as the formation of darker products with lower colour saturation. In the turmeric-containing system b* decreased from 60.57 to 28.16, whereas in the madder-containing system b* increased from 59.11 to 83.83. These differences may be associated with the distinct photochemical behaviour of anthraquinone colorants and curcuminoids, as well as possible photooxidative changes in the epoxy matrix [56,68,70].

*Changes in the hue angle (h°)*

The initial hue angle was 99.41°, corresponding to the yellow–green region. After only 15 s, a distinct shift to 84.87° occurred, indicating a transition toward a more yellow–orange hue. In the range 1.5–6 min, h° remained relatively stable (85–89°), which is consistent with the observed plateau for L* and the moderate changes in a* and b*. After 8–10 min, h° decreased to 77.79–77.53°, indicating a clear shift toward the orange-brown region, in agreement with the increase in a* and the pronounced decrease in b*.

### 2.4. Results of Colour Measurements for the Polyester Coating Formulation Containing Madder

**Figure 17:**
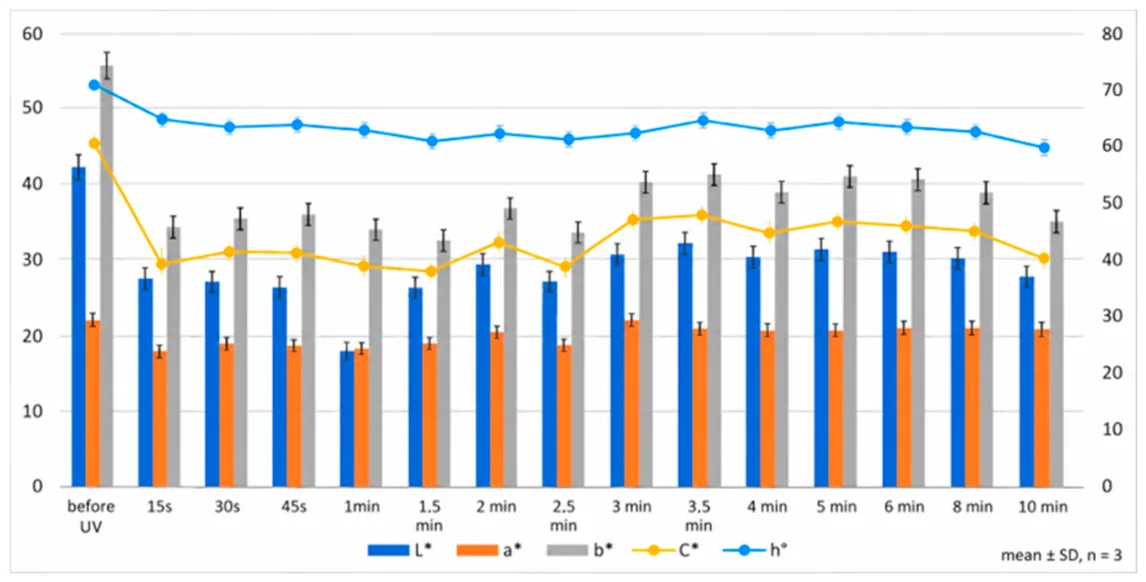
Changes in the colour coordinates L*, a*, and b*, as well as chroma (C*) and hue angle (h°), for the polyester coating formulation containing madder during UVB exposure from 15 s to 10 min.

The results of the colour measurements for the polyester coating formulation containing madder, recorded before and after UV irradiation, are presented in Figure 17.

The initial state of the formulation (before irradiation) was characterized by moderate lightness (L* = 42.05), a positive a* value (21.98), and a high b* value (55.56), indicating a distinct yellow-orange colour. The high chroma (C* = 59.75) and hue angle (h° = 68.41°) confirm the dominance of orange tones with a significant contribution of the yellow component.

Changes in lightness (L*)

A decrease in lightness was observed already after 15 s of UVB exposure, with L* dropping from 42.05 to 27.66, indicating darkening of the sample. The strongest decrease in L* occurred after 1 min (L* = 18.16), corresponding to a reduction in more than 50% relative to the initial value. During the subsequent course of UV exposure (1.5–6 min), L* partially increased and stabilized in the range of 28–32, which may indicate reorganization of the sample structure after the initial destabilization. In the final stage (8–10 min), the lightness remained at the level of 28–31 and did not return to the initial value. This indicates permanent darkening of the system as a result of UVB irradiation.

Changes along the red–green axis (a*)

The a* parameter decreased from 21.98 (before UV) to 17.67 after 15 s, indicating a reduction in the contribution of the red component. In the following minutes, a* values fluctuated within the range of 18–23, without a clear monotonic trend. In contrast to the sample containing the epoxy resin, no systematic disappearance of the red component was observed; the a* parameter remained positive throughout the entire exposure period, indicating retention of the orange-red character despite UV irradiation.

Changes in the yellow component (b*)

The most significant changes concern the b* parameter. After only 15 s, b* decreased from 55.56 to 34.46, indicating a rapid reduction in the intensity of the yellow chromophore. The lowest values were observed between 1 and 2.5 min of irradiation (b* in the range of 32–37). In the later stage of exposure (3–6 min), a partial increase in b* to approximately 40–42 occurred; however, this value did not reach the initial level. After 10 min, b* was 33.96, indicating a permanent reduction in the yellow colour by approximately 40% relative to the initial state.

Colour saturation (C*)

The C* parameter showed a significant decrease already after 15 s (from 59.75 to 38.72), indicating pronounced desaturation of the colour. After the following minutes of irradiation, C* fluctuated in the range of 38–47, without reaching the initial level. The final value after 10 min (C* = 39.92) confirms the persistent reduction in colour saturation as a result of UV exposure.

*Changes in the hue angle (h°)*

The hue angle decreased from 68.41° to approximately 62° already after 15–45 s, indicating a shift toward a more reddish hue. During the subsequent course of exposure, h° remained relatively stable (60–63°), with a slight decrease to 58.28° after 10 min. This indicates a shift in colour toward a more red-orange region, accompanied by a simultaneous reduction in the contribution of the yellow component.

The CIELAB colourimetric analysis (D65/10°) clearly demonstrated that exposure to UVB radiation causes significant and differentiated colour changes in the investigated systems (K + E + Photo, M + E + Photo, and M + P + Photo), affecting lightness (L*), chromatic coordinates (a*, b*), as well as the derived parameters (C*, h°). The observed changes indicate differences in both the mechanism and the rate of optical transformations depending on the composition of the system. In the case of the turmeric-containing sample, the dominant effect was a process leading to darkening and decolouration. Already within the first seconds, a sharp decrease in lightness and a distinct change in the a* coordinate were observed, indicating a shift from greenish to reddish tones. During further irradiation, a continued decrease in the colour parameter L* was noted, corresponding to a transition toward dark, weakly saturated tones. In the period from 0.5 to 6 min of irradiation, the colour parameters showed relative stabilization with moderate fluctuations. After 8–10 min, strong darkening, pronounced desaturation, and a shift in hue toward the orange-brown region were observed. These trends are consistent with the structural transformations identified by EPR and FTIR. For the sample containing madder in the epoxy resin, a short initial destabilization phase (decrease in L*, a*, and b*) was followed by a gradual colour change leading to brightening and an increase in the contribution of the yellow component. With increasing exposure time, a systematic decrease in a* and increases in b* and C* were observed, while the hue angle h° shifted toward a more yellow region. After 8–10 min of irradiation, the parameters indicated a lighter and more saturated colour with a dominant yellow contribution. This behaviour suggests that, in this system, UVB does not lead to typical “browning” but rather changes the proportions of the colour components, resulting in a shift in tone toward the yellow-orange region. A different behaviour of madder was observed in the sample containing the polyester resin. The key phenomenon was gradual darkening during the initial stage of irradiation, accompanied by a strong decrease in b* and C* (desaturation). At longer exposure times, the values partially stabilized; however, even after 10 min the system did not return to its initial state. The final effect was a darker, less saturated colour shifted toward the red-orange region.

A critical comparison of the three systems shows that the colour response cannot be attributed to UV exposure alone, because the direction and rate of the changes depend on the natural colorant, polymer matrix, photoinitiator, and polymerization mechanism. The different behaviour of madder in the epoxy and polyester coating formulations is particularly important in this respect: the same colorant shows different temporal changes in the CIELAB parameters, consistent with the different changes observed by EPR and FTIR. Thus, the colour measurements support the spectroscopic observations, but they should be regarded as a macroscopic response of the complete coating formulation rather than as a direct measure of a single chemical process.

## 3. Materials and Methods

### 3.1. Reagents and Materials

For sample preparation, natural plant-derived colorants as well as standard photoinitiators and resins were used: Madder (*Rubia tinctorum*) extract (OSL Color Lab, Szczytna, Poland); Turmeric (*Curcuma longa*) powder (country of origin: India; Kania, Poland); Radical photoinitiator: Irgacure 651 (2,2-dimethoxy-2-phenylacetophenone, 98%, Sigma-Aldrich, St. Louis, MO, USA); Cationic photoinitiator: triarylsulfonium hexafluoroantimonate, 50% solution in propylene carbonate (Sigma-Aldrich, St. Louis, MO, USA); Polimal^®^ 109 unsaturated polyester resin (Sarzyna Chemical, Nowa Sarzyna, Poland); and Epidian^®^ 5 low-molecular-weight epoxy resin (Sarzyna Chemical, Nowa Sarzyna, Poland).

### 3.2. Sample Preparation

#### 3.2.1. Preparation of Colorant–Photoinitiator Mixtures

Madder and turmeric were each dispersed with the cationic photoinitiator triarylsulfonium hexafluoroantimonate at a weight ratio of 10:0.02.

#### 3.2.2. Preparation of Colorant–Photoinitiator–Resin Mixtures

Three coating formulations were prepared. Formulation 1 contained madder extract (0.5 g), the ionic photoinitiator triarylsulfonium hexafluoroantimonate (0.02 g), and Epidian 5 epoxy resin (9.48 g). All components were dispersed together using a disperser equipped with a dispersing disc. Formulation 2 contained turmeric (0.5 g), the ionic photoinitiator triarylsulfonium hexafluoroantimonate (0.02 g), and Epidian 5 epoxy resin (9.48 g). In all three formulations, the components were dispersed together using a disperser equipped with a dispersing disc.

### 3.3. UV Irradiation Conditions

Paint samples placed in glass capillaries and deposited on glass slides were irradiated using an EMITA VP-60 medium-pressure mercury UV lamp (Famed-1, Łódź, Poland) with a nominal electrical power of 180 W. According to the published spectral characteristics of the lamp’s mercury emitter, its emission covers a broad wavelength range of approximately 230–580 nm, with prominent ultraviolet emission lines at 265, 297, 302, 313, and 366 nm. The emission lines at 297, 302, and 313 nm fall within the UVB spectral range, while the line at 366 nm corresponds to UVA radiation. Therefore, the UVB designation used in this study refers to the UVB component of the lamp’s polychromatic emission rather than to monochromatic irradiation at 320 nm. The irradiation time for each sample was controlled and ranged from 0 to 10 min.

The samples were positioned at a distance of approximately 5 cm from the UV emitter. The UV irradiance at the sample surface was 0.658 mW/cm^2^.

Immediately after each irradiation stage, the capillaries were transferred to the EPR spectrometer, where spectra were recorded in order to capture the time-dependent changes in the paramagnetic signals. The samples deposited on glass slides were subjected to FTIR analysis, and colour measurements were also performed.

### 3.4. EPR Measurements

**Figure 18:**
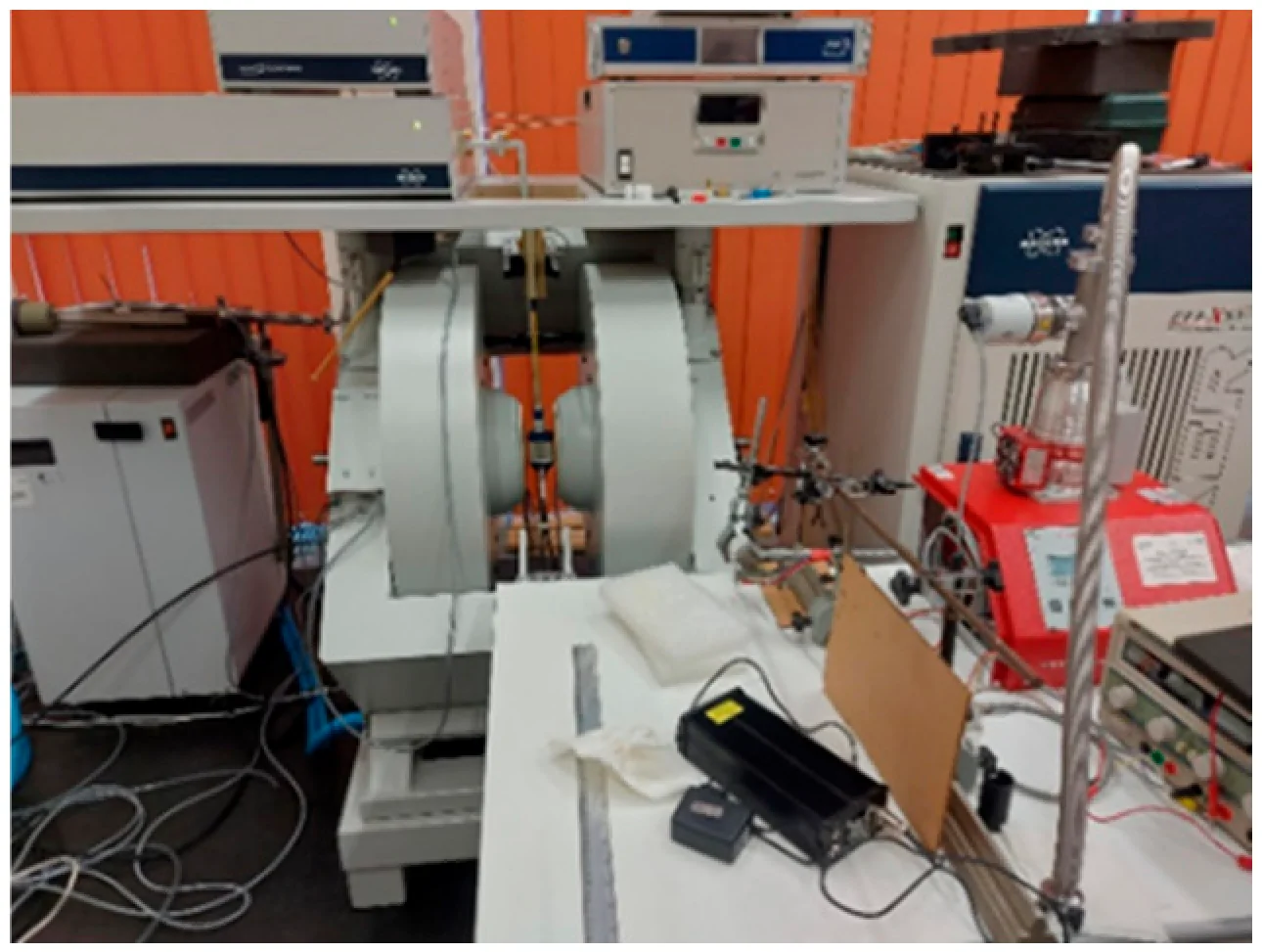
EPR measurement system (Bruker ELEXSYS) used to record the spectra of the samples before and after UV irradiation.

EPR spectra were recorded using a Bruker ELEXSYS E680 spectrometer (Figure 18). The analyses were carried out at room temperature. Electron paramagnetic resonance (EPR) was used to monitor the formation and decay of free radicals in the investigated samples during UV irradiation.

The recorded EPR spectra were processed to determine the basic spectroscopic parameters. The spectroscopic g-factor was calculated from the resonance condition according to the following equation [5,10,71]:

$$g=\frac{\mathrm{hv}}{{\mu_{B}B}_{0}}$$

where h is Planck’s constant, ν is the microwave frequency (GHz), *μB* is the Bohr magneton, and B0 is the resonance field [G].

The estimated uncertainty in the determination of the g factor was ±0.003 under the applied experimental conditions.

### 3.5. FTIR Measurements

The FT-IR spectra of the cured coatings were recorded using a Thermo Scientific Nicolet 6700 FT-IR spectrophotometer (Thermo Fisher Scientific, Waltham, MA, USA) equipped with a helium–neon (HeNe) laser in the range of 600–4000 cm^−1^ with a resolution of 4 cm^−1^. The data are presented as transmittance (%) versus wavenumber (cm^−1^).

### 3.6. Colour Measurements

Color parameters were determined using a Test-An 45/0 spectrophotometer (Anticorr, Gdańsk, Poland) in accordance with ISO 11664-4:2019 [72]. Measurements were performed under directional illumination at 45° ± 5°, using a CIE D65 standard illuminant in SCE (Specular Component Excluded) mode with a spectral interval of 10 nm. Color evaluation was based on the CIELAB color space parameters (L*, a*, b*).

where

L*—lightness (from 0 = black to 100 = white),

a*—green-red axis (from −60 to green, + 60 = red),

b*—blue-yellow axis (from −60 = blue to + 60 = yellow),

C*—saturation (≥0),

h°—hue angle (from 0° to 360°).

Chroma (saturation) C* is the distance of a point from the origin of the coordinate system (the L* lightness axis). It is calculated using the Pythagorean theorem based on the a∗ and b∗ coordinates:

$$C^{*}=\;\sqrt{{\left(a^{*}\right)}^{2}+{\left(b^{*}\right)}^{2}}$$

The hue angle h° defines the direction of the vector in the a*b* plane. It is expressed in degrees, ranging from 0° to 360°. It is calculated using the inverse tangent function:

$$h^{{}^{\circ}}=\arctan\;(\frac{b^{*}}{a^{*}})$$

## 4. Conclusions

The combined EPR, FTIR, and CIELAB analyses demonstrated that UV irradiation induces distinct changes in the colour and chemical structure of polymer coating formulations containing turmeric and madder. EPR measurements revealed changes in paramagnetic signals, including broad components tentatively attributed to transition-metal centres and narrow signals consistent with organic radicals. FTIR analysis showed changes in characteristic absorption bands, supporting the occurrence of photochemical transformations. The formation of phenoxyl, semiquinone, and oxygen-centred radicals is proposed as a possible reaction pathway, although their exact structures could not be unambiguously identified.

The colour response depended on the formulation and irradiation time. Turmeric-containing epoxy coatings exhibited pronounced darkening and loss of colour saturation, whereas madder-containing epoxy coatings showed a shift towards lighter, more yellow tones. Changes in the polyester formulation containing madder developed more slowly. These differences may result from the combined effects of polymer matrix, photoinitiator, and polymerization mechanism. The different UV responses of the investigated formulations may also be influenced by their photoinitiation mechanisms. In the epoxy systems, triarylsulfonium hexafluoroantimonate generates strong acids upon UV exposure, initiating cationic ring-opening polymerization. In contrast, Irgacure 651 generates radicals that initiate polymerization of the unsaturated polyester system. These distinct reaction environments may contribute to the different rates of spectral and colour changes observed during irradiation. However, their individual effects on colorant degradation cannot be isolated from those of the polymer matrices under the present experimental conditions.

The results provide a basis for further development of UV-based technologies for producing coatings with controlled colour effects using natural colorants, including the potential design of dedicated UV irradiation devices. The feasibility and reproducibility of such applications require further investigation. Other environmental conditions and irradiation methods, including humidity, sunlight, LED, laser, and electron-beam irradiation, remain potential directions for future research.

## Figures and Tables

**Table 1:** EPR signal parameters of madder extract before and after UV irradiation.

Sample	ν [GHz]	Intensity [a.u]	B0 (Br)	Bpp	g
**Line A**					
Madder + UV	9.441	0.3048	1585	65.94	4.25
Madder (without UV)	9.446	0.3963	1586	64.65	4.25
**Line B**					
Madder + UV	9.441	0.8839	3072	302.34	2.19
Madder (without UV)	9.446	no identification			
**Line C**					
Madder + UV	9.441	no identification			
Madder (without UV)	9.446	no identification			
**Line D**					
Madder + UV	9.441	5.9171	3370	6.83 (S)	2.001
Madder (without UV)	9.446	4.6483	3372	6.137	2.001

**Table 2:** EPR signal parameters of ground turmeric before and after UV irradiation.

Sample	ν [GHz]	Intensity [a.u]	B0 (Br)	Bpp	g
**Line A**					
Turmeric + UV	9.860055	0.2042	2409	38.18	2.92
Turmeric (without UV)	9.86052	-	-	-	-
**Line B**					
Turmeric + UV	9.860055	1.32244	3404	569.89	2.06
Turmeric (without UV)	9.86052	0.6711	3444	675.93	2.04

**Table 3:** EPR spectral parameters of the sample before UV irradiation.

Region	ν [GHz]	B_0_ [G]	Bpp [G]	I [a.u.]	g
**Line A**					
	9.448916	1469	1101	0.5678	4.59
**Line B**					
**B-1**	9.448916	2298	63.79	0.0216	2.93
**B-2**	9.448916	2551	164.36	0.0746	2.64
**B-3**	9.448916	2907	181.95	0.4296	2.32
**Line C**					
	9.448916	3372	5.75	0.8609	2.001

**Table 4:** Simplified summary of the EPR signal parameters for the coating formulation containing madder, Epidian^®^ 5, and a cationic photoinitiator during UV irradiation.

UV Time [Min]	Line A—Intensity [a.u.]	Line A—Bpp [G]	Line B (B-3)—B_0_ [G]	Line B (B-3)—Bpp [G]	Line B (B-1 + B-2 + B-3)—Intensity [a.u.]	Line C—Intensity [a.u.]	Line C—Bpp [G]
0.5	0.2053	1309	2902	151.25	0.6985	0.8226	6.36
1	0.6898	1331	2911	150.73	0.7327	0.8908	6.33
2	0.52829	1181	2915	157.43	0.78013	0.88989	6.37
3	0.4866	1237	2904	148.4	0.53619	0.85279	6.42
4	1.2119	1973	2917	176.58	0.865	0.9673	6.6193
5	0.2886	1193	2904	136.24	0.6196	0.9264	6.7251
6	0.67032	1904	2907	158.05	0.67321	0.9123	6.649
8	0.485	1646	2912	170.25	0.66618	0.9747	6.97
10	0.2329	1101	2910	134.62	0.72113	1.136	7.17

**Table 5:** EPR signal parameters for the coating formulation containing turmeric, Epidian^®^ 5, and a cationic photoinitiator before and after UV irradiation.

Coating Formulation: Turmeric + Epidian^®^ 5 + Cationic Photoinitiator	Line	ν [GHz]	Intensity [a.u]	B_0_ (Br) [G]	Bpp [G]	g
Before UV irradiation	B	9.448228	0.9374	2983	214.11	2.26
0.5 min UV	B	9.452998	0.9339	2972	212.33	2.27
1 min UV	B	9.453671	0.6886	2975	208.7	2.27
1.5 min UV	B	9.451384	0.7963	2976	208.88	2.26
2 min UV	B	9.452145	0.7367	2973	203.55	2.27
2.5 min UV	A	9.451022	0.1907	2287	78.88	2.95
	B	9.451022	0.8742	2974	204.86	2.27
3 min UV	B	9.451653	0.804	2981	214.05	2.26
3.5 min UV	B	9.451662	0.8312	2976	213.78	2.26
4 min UV	B	9.45371	0.7432	2978	220.94	2.26
5 min UV	A	9.45371	0.06102	2450	104.72	2.75
	B	9.45371	0.7877	2981	212.86	2.26
6 min UV	B	9.453702	0.6945	2984	197.58	2.26
7 min UV	B	9.452981	0.7336	2988	198.79	2.25
8 min UV	B	9.453955	0.6998	2983	202.13	2.26
10 min UV	B	9.45064	0.7237	2989	198.51	2.25

**Table 6:** EPR spectral parameters of the sample before UV irradiation.

Coating Formulation: Madder + Polimal^®^ 109 + Irgacure 651	Line	ν [GHz]	Intensity [a.u]	B_0_ (Br) [G]	Bpp [G]	g
Before UV irradiation	A	9.449899	0.04966	1584.5	58.82	4.26
	B	9.449899	0.56019	2961	202.42	2.28
	C	9.449899	0.1085	3343	212	2.02
	D	9.449899	0.4648	3374	5.9636	2.001

**Table 7:** EPR signal parameters of madder-containing samples as a function of UV irradiation time (0–10 min).

UV Irradiation Time (Madder + Polimal^®^ 109 + Irgacure 651)	Line	ν [GHz]	Intensity [a.u]	B_0_ (Br) [G]	Bpp [G]	g
Before UV irradiation	A	9.449899	0.04966	1584.5	58.82	4.26
	B	9.449899	0.56019	2961	202.42	2.28
	C	9.449899	0.1085	3343	212	2.02
	D	9.449899	0.4648	3374	5.9636	2.001
0.5 min UV	A	9.451	0.0445	1588	80.38	4.25
	B	9.451	0.5089	2958	207.71	2.28
	C	9.451	0.00779	3301	169.05	2.04
	D	9.451	0.4182	3374	6.27	2.001
1 min UV	A	9.45217	0.047	1584.5	51.99	4.26
	B	9.45217	0.2792	2913.1	199.16	2.31
	C	9.45217	0.0255	3263	70.209	2.06
	D	9.45217	0.4723	3373.68	6.14	2.001
2 min UV	A	9.450	0.04972	1586	53.56	4.25
	B	9.450	0.2911	2928	315.9	2.3
	C	9.450	0.004632	3237	30.66	2.08
	D	9.450	0.496679	3373	6.67	2.001
3 min UV	A	9.452661	0.0422	1596.3	58.92	4.23
	B1	9.452661	0.037	2861	108	2.36
	B2	9.452661	0.047736	2857	77.55	2.36
	C	9.452661	0.0681	2995	68.07	2.25
	D	9.452661	0.50723	3374	6.43	2.001
4 min UV	A	9.452055	0.0352	1577	43.13	4.27
	B	9.452055	0.6138	2857	73.54	2.36
	C	9.452055	0.0975	3198	136.4	2.11
	D	9.452055	0.4906	3373.68	7.37	2.001
5 min UV	A	9.454100	0.04914	1585	72	4.26
	B	9.454100	0.40835	2849	70.13	2.36
	C	9.454100	0.00351	2982	51.9	2.26
	D	9.454100	0.4654	3375	6.85	2.001
6 min UV	A	9.451592	0.0411	1585	61.96	4.25
	B	9.451592	0.6008	2853	79.54	2.36
	C	9.451592	0.1225	3095	188	2.18
	D	9.451592	0.0477	3373	7.38	2.001
8 min UV	A	9.454802	0.0359	1587.1	71.53	4.25
	B	9.454802	0.4034	2856	61.89	2.36
	C	9.454802	0.0058	3066	175.9	2.2
	D	9.454802	0.44012	3375	8.75	2.001
10 min UV	A	9.452293	0.055	1595.5	78.28	4.27
	B	9.452293	0.2956	2855	98.06	2.36
	C	9.452293	0.0373	3106	195.57	2.17
	D	9.452293	0.5127	3374	7.009	2.001

## Data Availability

The original contributions presented in this study are included in the article. Further inquiries can be directed to the corresponding authors.
